# The neglected contexts and outcomes of evidence-based management: a systematic scoping review in hospital settings

**DOI:** 10.1108/JHOM-03-2021-0101

**Published:** 2021-12-28

**Authors:** Tina Sahakian, Lina Daouk-Öyry, Brigitte Kroon, Dorien T.A.M. Kooij, Mohamad Alameddine

**Affiliations:** Evidence-based Healthcare Management Unit, American University of Beirut Medical Center , Beirut, Lebanon; Suliman S Olayan School of Business , American University of Beirut , Beirut, Lebanon; Department of Human Resource Studies, Tilburg School of Social and Behavioral Sciences , Tilburg University , Tilburg, The Netherlands; College of Health Sciences , University of Sharjah , Sharjah, United Arab Emirates

**Keywords:** Systematic scoping review, Evidence-based management (EBMgt), Healthcare management, Management decision-making, Healthcare managers, Hospitals

## Abstract

**Purpose:**

The coronavirus disease 2019 (COVID-19) pandemic highlighted the necessity of practicing Evidence-based Management (EBMgt) as an approach to decision-making in hospital settings. The literature, however, provides limited insight into the process of EBMgt and its contextual nuances. Such insight is critical for better leveraging EBMgt in practice. Therefore, the authors' aim was to integrate the literature on the process of EBMgt in hospital settings, identify the gaps in knowledge and delineate areas for future research.

**Design/methodology/approach:**

The authors conducted a systematic scoping review using an innovative methodology that involved two systematic searches. First using EBMgt terminology and second using terminology associated with the EBMgt concept, which the authors derived from the first search.

**Findings:**

The authors identified 218 relevant articles, which using content analysis, they mapped onto the grounded model of the EBMgt process; a novel model of the EBMgt process developed by Sahakian and colleagues. The authors found that the English language literature provides limited insight into the role of managers' perceptions and motives in EBMgt, the practice of EBMgt in Global South countries, and the outcomes of EBMgt. Overall, this study’s findings indicated that aspects of the decision-maker, context and outcomes have been neglected in EBMgt.

**Originality/value:**

The authors contributed to the EBMgt literature by identifying these gaps and proposing future research areas and to the systematic review literature by developing a novel scoping review method.

## Introduction

The coronavirus disease 2019 (COVID-19) pandemic highlighted the necessity of using data to inform healthcare decision-making. To face the extraordinary patient care challenges COVID-19 caused, medical professionals are relying not only on the existing scientific literature and their clinical judgment, but also on emergent data about the virus ( del Rio and Malani, 2020 ). In parallel, to face the extraordinary operational challenges, hospital managers must combine their experience with existing research, as well as, emergent data about managing the virus ( Adams and Walls, 2020 ). Additionally, since the organization of healthcare delivery differs across and within countries and healthcare organizations ( Anell and Willis, 2000 ), managers must consider data in concert with contextual factors, like resources, culture and laws, and must tailor solutions accordingly ( Tanne *et al.* , 2020 ). Ultimately, since this pandemic highlighted the necessity of using data and contextualizing it to inform decision-making, it put evidence-based management (EBMgt) at the forefront of hospital management.

EBMgt involves gathering evidence from different sources, appraising its quality and using it to inform decisions ( Barends *et al.* , 2014 ). By encouraging the use of critically appraised evidence, EBMgt aims to improve decisions, and consequently organizational outcomes ( Barends *et al.* , 2014 ). This is an opportune time to integrate current knowledge about EBMgt in hospital settings into a unifying framework and identify the gaps in it. Doing so will allow us to better understand EBMgt and leverage it in practice. Accordingly, our aim is to integrate the literature on the EBMgt process in hospital settings, identify the gaps and outline areas for future research. We conducted a systematic scoping review of the EBMgt literature in hospital settings, and analyzed the results using a novel framework of the EBMgt process. Unlike systematic reviews, systematic scoping reviews are more comprehensive and their aim is to map the existing literature on a topic rather than synthesize it to answer a specific question ( Levac *et al.* , 2020 ).

### Systematic reviews of EBMgt in healthcare

Systematic reviews of the EBMgt literature have concluded that the literature offers limited empirical insight into how EBMgt is implemented in different contexts ( Currie, 2013 ; Reay *et al.* , 2009 ; Rynes and Bartunek, 2017 ). Within healthcare, systematic reviews of EBMgt have examined its components in isolation rather than exploring the process through which it is implemented. For example, Jaana *et al.* (2014) examined the availability and accessibility of systematic reviews and meta-analyses for healthcare managers. They found that reviews addressing purely management-related topics (e.g. pay-for-performance) were rare and challenging to retrieve. Roshanghalb *et al.* (2018) identified the decision-makers the EBMgt literature has focused on, the decisions they make and evidence they use. They found that the literature has focused on hospital managers and medical professionals, who rely on expert opinion, organizational evidence and research to make different decisions, including performance assessment and strategic planning. Sarkies *et al.* (2017) examined the effectiveness of implementation strategies meant to improve research evidence uptake in decision-making. They found various strategies and identified factors that contributed to their effectiveness. These systematic reviews provide insight into some aspects of EBMgt, but not into the process of EBMgt practice and the contextual factors that influence it. Such insight is necessary since evidence availability alone does not guarantee its use, rather evidence use is influenced by contextual factors ( Baba and HakemZadeh, 2012 ). Additionally, while these reviews make recommendations for future research, they do not identify the gaps in our knowledge of the EBMgt process in hospital settings.

As such, our aim in this study is to integrate the literature on EBMgt process in hospital settings into a unifying framework, identify the gaps and outline core areas for future research. We explored the following research questions: (1) What aspects of the EBMgt process in hospital settings have been studied? (2) What are the main gaps in our knowledge of the EBMgt process in hospital settings? and (3) How generalizable is the literature to different contexts? Answering these questions will deepen our understanding of how managers can use evidence for decision-making, both in daily practice and to face global healthcare challenges.

To answer our questions, we conducted a systematic scoping review of the EBMgt literature in hospital settings. We decided to analyze the results from a process perspective ( Pettigrew, 1992 ). This is suitable since EBMgt is an approach to *decision-making* : the deliberation process before, during and after choosing a course of action ( Elwyn and Miron-Shatz, 2009 ) and since existing systematic reviews have not provided insight into *the EBMgt decision-making process* .

### Theoretical framing

The process perspective examines phenomena as dynamic sequences of actions that develop and change over time ( Pettigrew, 1992 ). Human actors construct processes through their actions, their subjective interpretations change processes, and they mobilize aspects of the context to obtain outcomes important to them. The process perspective also highlights the importance of structures, as the context in which actions occur, and which both shape and are shaped by actions ( Pettigrew, 1992 ; Pettigrew *et al.* , 2001 ). To guide our analysis, we juxtaposed three EBMgt frameworks in healthcare that present EBMgt as a process ( Table 1 ). Kovner and Rundall (2006) conceptualized EBMgt as a linear decision-making process with a series of steps, where evidence can be incorporated in the steps of analyzing alternatives and selecting an alternative. This model assumes that managers are rational; they make a complete search of all alternatives and make decisions based on organizational goals ( Langley *et al.* , 1995 ), and does not account for the impact of context ( Dean and Bowen, 1994 ). Taking a bounded rationality approach, Baba and HakemZadeh (2012) conceptualized EBMgt, a process executed at the individual level, and influenced by individual, organizational and institutional factors. These factors influence the evidence that managers use, the alternatives they generate and their choice between alternatives. While Baba and HakemZadeh (2012) developed this model based on the extant literature, it does not explicate the details of the EBMgt process.

**Figure 1:**
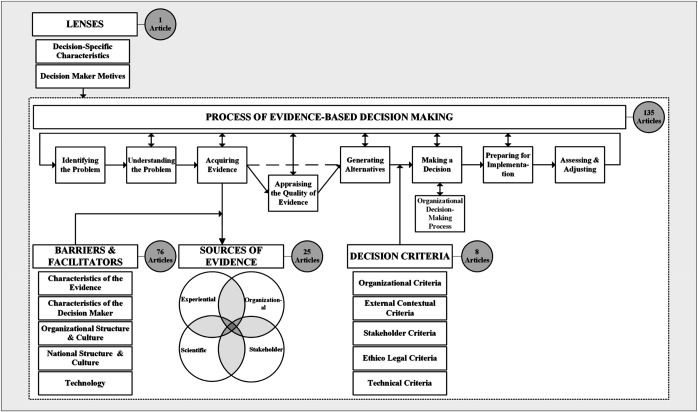
Sahakian (2020) Grounded Model of the EBMgt Process and Distribution of the Scoping Review Articles across its Dimensions

Sahakian’s (2020) Grounded Model of the EBMgt Process (reproduced with permission, Figure 1 ) is an empirically-driven model of the evidence-based decision-making process and its contextual nuances in hospitals. It explicates, using empirical data, Baba and HakemZadeh’s (2012) proposed model and integrates elements of Kovner and Rundall's (2006) stepwise process. It includes five dimensions: Process of Evidence-based Decision-making, Sources of Evidence, Barriers and Facilitators, Decision Criteria and Lenses. The Process involves a series of eight steps, among these are the steps of acquiring evidence and appraising the quality of the evidence, which are the hallmarks of EBMgt ( Barends *et al.* , 2014 ). At the step of acquiring evidence, four Sources of Evidence are identified: experiential, organizational, scientific and stakeholder. Managers' progress through the steps is influenced by individual, organizational and national factors. The dimensions of Barriers and Facilitators, Decision Criteria and Lenses, capture these factors and their influence on the process. Barriers and Facilitators refer to factors that hinder or help evidence acquisition and use. Decision Criteria refer to contextual conditions that must be balanced alongside the evidence when deciding between decision alternatives. Finally, Lenses refer to managers' motives and perception, which influence their decision-making process.

We decided to adopt the Grounded Model of the EBMgt Process (hereafter the Model) to guide our analysis for several reasons. First, the Model depicts the EBMgt process and the different contextual factors that influence it. Second, it pinpoints when and how these contextual factors impact the process. Finally, it was developed empirically. While the data were collected in hospitals in Lebanon; a middle-income Global South country in the Middle East, the Model is nonetheless embedded in the larger EBMgt literature with many of its themes overlapping with the literature ( Sahakian, 2020 ).

## Methods

We conducted a scoping review on the topic of EBMgt in hospital settings. We searched four online databases: PubMed, CINAHL, PsycINFO and Cochrane Library, for peer reviewed, English-only journal articles without restrictions on publication year. We describe our search strategy briefly below, and in detail in Supplementary File 1 .

Since the EBMgt literature is spread across several fields and does not strictly use EBMgt terminology ( Briner *et al.* , 2009 ), we developed a novel review methodology. It involved two systematic searches; first using EBMgt terminology, and second using terminology associated with the EBMgt concept. Our method is novel, compared to the traditional scoping reviews, because we used the results of the first search to derive keywords for the second search. This resulted in a four-step methodology, which we implemented from April 2015 to October 2019.

### General systematic search

First, we searched the four databases using the key-terms “evidence-based” OR “evidence-driven” OR “evidence-informed” AND “healthcare” AND “management.” These terms were included in the search since they often refer to similar concepts, though with some narrow variations in their definition (see Bowen *et al.* , 2009 ; Nevo and Slonim-Nevo, 2011 ).

We removed duplicates and applied four filters to narrow down the articles. The first two filters involved removing titles that reflected clinical (e.g. alginate dressings for leg ulcers) and irrelevant (e.g. Second World War repression) topics. The third filter involved reading article abstracts and the fourth reading article full-texts and keeping articles that revolved around: *non-clinical evidence-based decision-making* by *managers* in (or including) *hospital settings* . Two researchers, including one of the authors, applied the filters separately and reconciled their differences after each filter. They also reviewed the reference lists of the remaining relevant articles.

### Keyword identification

Second, we extracted the keywords from the remaining relevant articles to identify EBMgt-related terminology. We developed an expert panel involving the researchers who applied the filters and two of the authors. The panel reviewed the relevance of the words to EBMgt and based on consensus decided on a list of 21 keywords (e.g. evidence-informed improvement; full list in Supplemental File 1 ).

### Keyword systematic search and reconciliation

Third, we searched the four databases using the 21 keywords and “healthcare” AND “management” NOT “clinical,” applying the same search criteria and filters as the first search. Fourth, we examined the overlap of the articles across the two searches and removed duplicates.

## Analysis

We analyzed the articles using a deductive content analysis approach ( Elo and Kyngas, 2008 ). The foundation for the categorization was the Model with its five dimensions encompassing 30 themes. Two of the authors conducted the categorization collaboratively by reading each article and assigning it to a relevant dimension(s) and theme(s). When articles did not fit the Model, they created new themes relying on inductive content analysis ( Elo and Kyngas, 2008 ). Engaging an independent coder, we assessed the reliability of our categorization (detailed in Supplemental File 1 ) and found moderate agreement, Cohen's *κ* = 0.59 (95% CI, 0.44 to 0.75).

## Results

**Figure 2:**
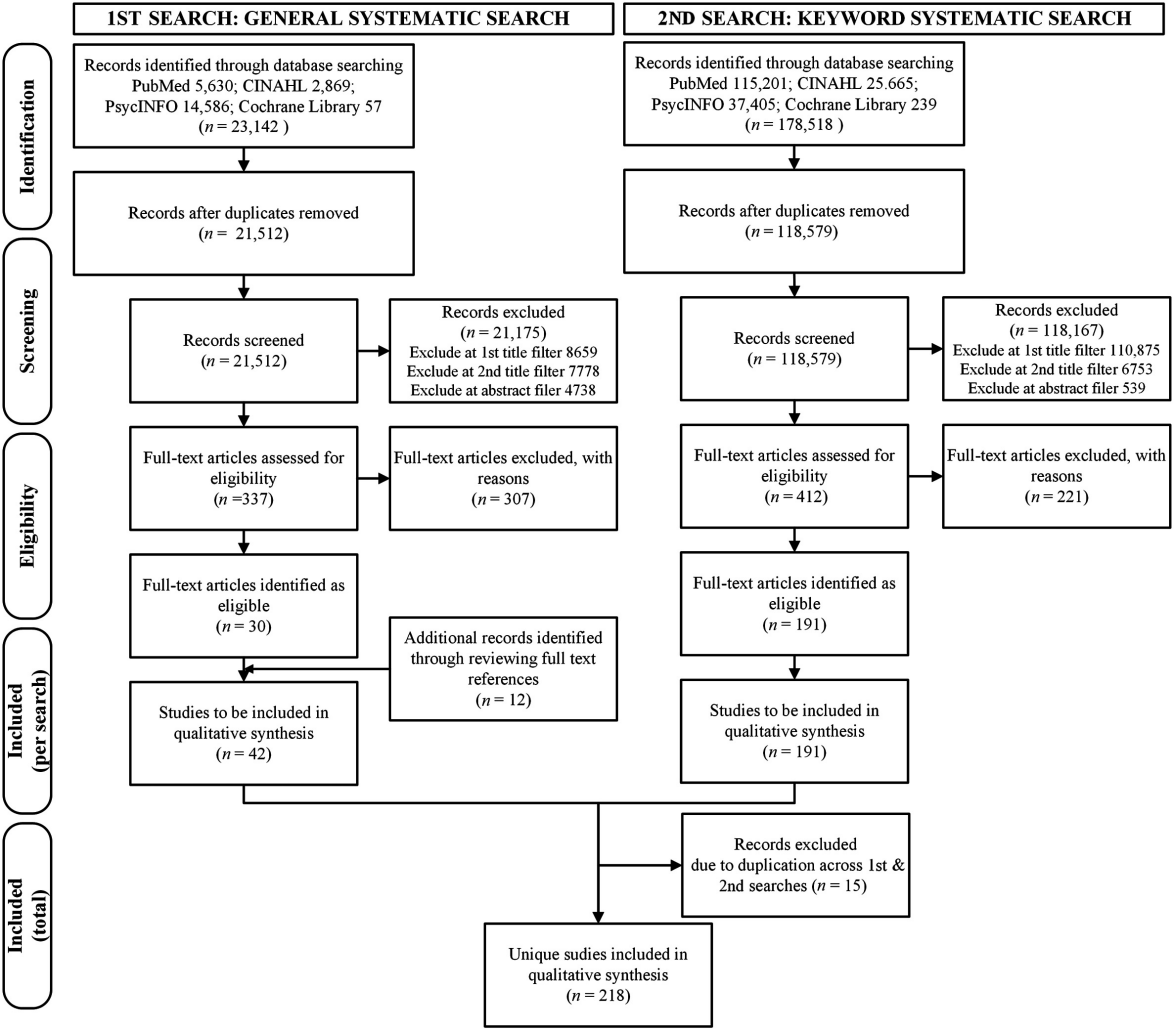
Search and filtering strategy results

The General systematic search yielded 23,142 articles and the Keyword systematic search 178,518 articles ( Figure 2 ). Applying the four filters, we identified 218 unique relevant articles (listed in Supplementary File 2 ). Agreement between the researchers applying the filters was high during the General systematic search, Cohen's *κ* = 0.85 (95% CI, 0.79 to 0.91) and moderate during the Keyword systematic search, Cohen's *κ* = 0.57 (95% CI, 0.48 to 0.66).

### Descriptive analysis

**Figure 3:**
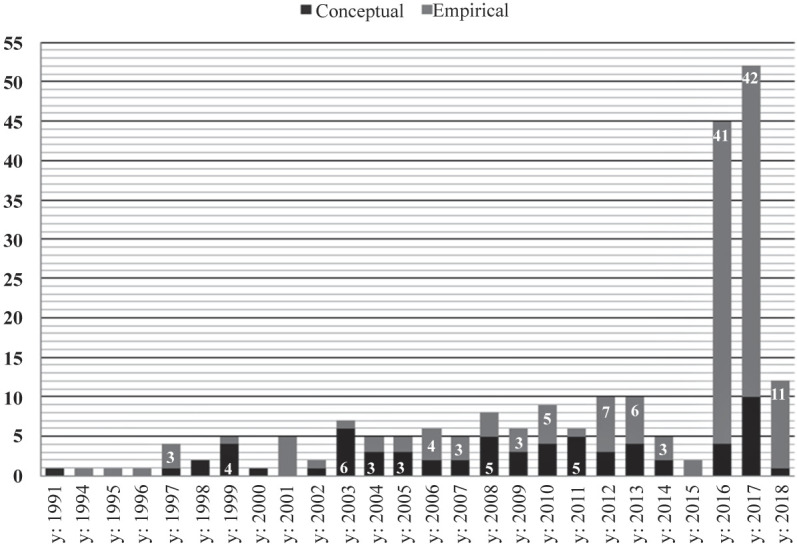
Frequency of articles over time and distribution of conceptual versus empirical articles

The first article was published in the year 1991 and the number of publications has increased steadily ( Figure 3 ). Examining the geographic distribution of studies, half were conducted in North America (50.00%) and another quarter in Europe (25.69%). Some studies were conducted in Australia (5.96%), Asia (5.50%) and the Middle East (5.50%), and only a few in South America (2.75%) and Africa (0.92%). Moreover, some studies were cross-cultural (3.67%). In North America, most studies were conducted in USA (37.61%), and in Europe, most were conducted in the UK (7.80%). Notably, the Global North countries of USA, Canada, Australia, New Zealand, UK, EU states, Russia, Israel, Japan, Singapore and South Korea accounted for 86.24% of all studies. Most articles were empirical in nature (68.81%), with the ratio of empirical to conceptual articles having increased over time ( Figure 3 ).

Examining the methodology of the empirical articles, half used quantitative methods (50.67%), the rest used qualitative (36.00%) and mixed methods (13.33%). They used a range of data collection methods ( Table 2 ), sometimes in-combination, including single-case studies (38.67%), interviews (31.33%) and cross-sectional surveys (25.33%). As for the conceptual articles, 17.65% were systematic reviews while the rest were literature reviews.

### Content analysis

Overall, the articles revolved around a range of hospital decisions, including financial, like resource allocation ( Baghbanian *et al.* , 2012 ), human resource, like nurse staffing ( Kullberg *et al.* , 2016 ), patient experience, like emergency department (ED) waiting times ( Wiler *et al.* , 2016 ), and information system decisions, like assessing the impact of electronic health records (EHR, Plantier *et al.* , 2017 ). We found that most of the articles could be categorized according to the five dimensions of the Model ( Figure 1 ), except for a handful of conceptual articles, which we categorized under a new theme “EBMgt concept.” We discuss some of the article under each dimension and theme below and summarize them all in the Supplementary Files 3–7 .

#### The process of evidence-based decision-making

We identified 135 articles (83.70% empirical) that either focus on a specific step of the eight-step EBMgt process or the overall process ( Supplementary File 3 ).

##### Specific step of the process

The articles focused on the steps of acquiring evidence, appraising the quality of the evidence, generating alternatives, making a decision, preparing for implementation, and assessing and adjusting. Among the steps that are core to EBMgt practice, acquiring evidence was studied extensively (45 articles). Some articles discussed strategies for evidence acquisition from different sources (strategies for searching the Internet, Kibbe *et al.* , 1997 ). Others discussed applied cases of evidence acquisition, like for an overcrowding problem in an ED ( Elamir, 2018 ), underlining the context-dependent nature of the evidence. Others collected evidence for specific problems (nursing burnout, Steege and Dykstra, 2016 ). In comparison to acquiring evidence, the step of appraising the quality of evidence received little attention (four articles). These articles mostly described strategies for appraising evidence quality (strategies for rating evidence strength, Lohr, 2004 ).

Among the steps that are not unique to EBMgt, while only a couple of studies focused on generating alternatives ( Elamir, 2018 ), and making a decision ( Testik *et al.* , 2017 ), many focused on preparing for implementation (13 articles) and assessing and adjusting (23 articles). The articles focusing on preparing for implementation were mostly applied cases. Some discussed tools to support implementation of evidence-based solutions, like simulations ( Gignon *et al.* , 2017 ). Others examined factors that influence implementation of different evidence-based solutions, like waiting time management initiatives ( Pomey *et al.* , 2013 ). The articles focusing on the step of assessing and adjusting were all applied cases evaluating the impact of implementing specific initiatives on specific outcomes (EHR on quality of care, Plantier *et al.* , 2017 ). The number of articles focusing on the steps of preparing for implementation and assessing and adjusting indicate that the success of EBMgt depends not only on the identification of evidence-driven solutions but also their successful implementation in the specific context of the organization.

##### The process overall

Some articles examined the EBMgt process among managers in hospitals ( Baghbanian *et al.* , 2012 ). Others developed tools, like digital platforms to support the application of the EBMgt process ( Gartnera and Padmanb, 2017 ). While others were applied cases describing the EBMgt process managers adopted to solve specific problems, like nurse staffing ( Kullberg *et al.* , 2016 ) and patient throughput ( Wiler *et al.* , 2016 ) in specific hospitals.

Overall, the EBMgt process steps identified in these articles overlapped considerably with each other and with the Model. It is worth noting that the step of aggregating the evidence, which is highlighted in some definitions of EBMgt ( Barends *et al.* , 2014 ) and was part of appraising the evidence in the Model, was identified by only one article ( Oetjen *et al.* , 2008 ). This brings into question how necessary this step is in the process. Moreover, the articles discussed a wide range of problems, many of which, like ED crowding ( Pines and Griffey, 2015 ), represent worldwide healthcare challenges, thus situating EBMgt at the core of tackling such pervasive problems.

#### Sources of evidence

This dimension refers to the sources from which managers acquire evidence. It includes experiential (i.e. experience, knowledge and judgment of practitioners), scientific (i.e. research literature), organizational (i.e. internal data) and stakeholder (i.e. stakeholders' input) evidence. We identified 25 articles (56% empirical) that fit under this dimension ( Supplementary File 4 ).

Among these articles, Råholm (2009) argued for a re-conceptualization of evidence. Others examined the evidence that managers use in practice and found that they combine various types of evidence from the four sources. To illustrate, Shoemaker *et al.* (2010) identified that for a hospital design decision, managers used literature searches (scientific evidence), consultation with architects (experiential evidence), financial costs (organizational evidence) and visits to other organizations (stakeholder evidence). While other articles argued for using specific types of evidence including specific types of scientific evidence (operations research, Capan *et al.* , 2017 ) and organizational evidence (patient experience data from twitter posts, Hawkins *et al.* , 2016 ). Overall, these studies highlight the different types of information that is available to hospital managers, and how they can use this information to inform decisions.

#### Barriers and facilitators

This dimension refers to factors that either hinder or help managers' acquisition and use of evidence. It includes the characteristics of the evidence (i.e. availability, appropriateness and time), the characteristics of the decision-maker (i.e. competencies and position), organizational structure and culture factors influencing accessing, capturing, and using evidence, national structure and culture, and technology. We identified 76 articles (53.95% empirical) that either attempted to identify all EBMgt barriers and facilitators or focused on one specific barrier or facilitator ( Supplementary File 5 ). We will discuss these articles according to the barriers and facilitators they identified from the Model.

Many articles identified characteristics of the evidence that hinder evidence use. As in the Model, articles found that evidence (un)availability (scarcity; Kovner *et al.* , 200 , or evidence overload; Liang *et al.* , 2012 ), evidence (in)appropriateness (poor quality; Gallego *et al.* , 2008 , and focus on Western contexts; Liang *et al.* , 2012 ), and the time consuming nature of acquiring evidence ( Ellen *et al.* , 2014 ) limits its acquisition. To overcome these barriers, articles suggested different methods to produce research evidence that fits managers' needs, like evidence co-creation ( Marshall, 2013 ).

Articles in the literature also found characteristics of the decision-maker that facilitate evidence use. Like the Model, they found that certain competencies, including data analysis knowledge, business knowledge and interpersonal skills, are necessary ( Niedzwiedzka, 2003 ; Kovner *et al.* , 2000 ) and discussed developing them through education and training ( Liang *et al.* , 2012 ; Finkler, 2002 ). Some articles also focused on managers' position in the organization, and how it can be used to promote evidence use ( Karamitri *et al.* , 2017 ). Moreover, the articles in the literature identified two additional facilitators, which were not part of the Model. Specifically, they found that certain demographic characteristics, like education level ( Jbilou *et al.* , 2007 ) and attitudes, like positive attitudes towards research are important for applying EBMgt ( Niedzwiedzka, 2003 ).

The articles also identified organizational structural and cultural factors that hinder or facilitate evidence use. As in the Model, the articles identified that organizational factors that facilitate accessing evidence (access to electronic databases; Niedzwiedzka, 2003 ), and capturing evidence (electronic medical records; Karamitri *et al.* , 2017 ) are necessary for evidence use. Adding to the Model, many articles found that organizational factors that facilitate producing evidence, like partnering with universities on research projects ( Jbilou *et al.* , 2007 ), and disseminating evidence, like establishing evidence dissemination units ( Ellen *et al.* , 2013 ), are important for evidence use. Finally, as in the Model, several articles found that several organizational factors that specifically emphasize using evidence are important. These factors included having a culture of evidence use, through incorporating EBMgt into the organizational mission ( Kovner and Rundall, 2006 ), and having leaders who model EBMgt behavior ( Karamitri *et al.* , 2017 ). These factors also included having human resource practices that promote EBMgt adoption, like offering EBMgt training programs ( Rundall *et al.* , 2007 ) and incorporating EBMgt into managers' performance appraisals ( Ellen *et al.* , 2013 ).

Finally, the articles found barriers and facilitators related to national structure. As in the Model, articles highlighted the importance of national cooperatives for research production that unite healthcare management researchers and practitioners ( Walshe and Rundall, 2001 ). Articles also included additional national structural factors not identified in the Model, like national information technology infrastructure ( Clancy and Cronin, 2005 ) and policy reforms to incentivize organizations ( Leatherman and Sutherland, 2007 ).

Overall, the barriers and facilitators to EBMgt are at the intersection of healthcare management research, education, practice and governance. Therefore, the responsibility of facilitating the adoption of EBMgt falls on these four groups working interdependently. Furthermore, despite the overlap between the themes in the articles and the Model, there were also discrepancies. The articles included some barriers and facilitators that are not part of the model, thus, the model could be amended to make it more representative of the overall literature. The Model also included some barriers and facilitators not found in the articles. The Model included national cultural barrier, referring to a national culture of sharing information, and specifically lack thereof in the Lebanese context where the Model was developed. The Model also included a standalone facilitator, technology, referring to increase in information availability resulting from overall advancement of healthcare technology, like EHR. This discrepancy might also be due to the national context where the model was developed where such technologies are not yet widespread ( Saleh *et al.* , 2016 ). We will explore the issue of national context in the discussion.

#### Decision criteria

This dimension refers to contextual conditions that are considered alongside the evidence when selecting between alternatives. Decision criteria are organizational (i.e. strategic plan, resources, culture and politics), external contextual (i.e. external systems, culture and politics), stakeholder interest and needs, ethicality and legality and technical (i.e. specialty-specific requirements). We found 8 articles (75% empirical) that either identified decision criteria, pinpointed when decision criteria impact the EBMgt process or both ( Supplementary File 6 ).

The articles identified a range of decision criteria, including organizational (e.g. resource considerations, Shoemaker *et al.* , 2010 ), stakeholder (patient safety, Gallego *et al.* , 2008 ), external contextual (external politics and marketing initiatives, Gallego *et al.* , 2008 ), ethics and technical medical considerations ( Baghbanian *et al.* , 2012 ). Moreover, while some ( Oetjen *et al.* , 2008 ) conceptualized that these criteria are defined once a problem is identified and are used to evaluate decision alternatives, others ( Baghbanian *et al.* , 2012 ) found that, in practice, these criteria are not predetermined early in the process. The latter finding is aligned with the Model and research showing that decision criteria are considered implicitly when choosing between alternatives ( Mintzberg *et al.* , 1976 ).

Interestingly, these criteria emerged when researchers were examining the EBMgt process in practice. This indicates the importance of focusing on context when examining EBMgt in practice. Moreover, two criteria identified in the articles, namely external funding and marketing initiatives, which we categorized as external contextual criteria, were not part of the Model. This suggests that the Model could be potentially amended to reflect the literature. Furthermore, the Model included criteria not identified in the literature, which suggests a potential gap in our knowledge on decision criteria and room for more research.

#### Lenses

This dimension refers to managers' motives and perceptions that impact how they make decisions, the evidence they use and the decision criteria they prioritize. Lenses differ from Barriers and Facilitators because they do not directly impact evidence acquisition; rather they impact other parts of the process or the process overall. Kyratsis *et al.* 's (2012) study, which proposed to explore managers' motives and determine “why different understandings and meanings emerge for one observation and how this explains different views of scientific evidence” (p. 5), could fall under this dimension. We will discuss the implication of finding only one article in this review that fits under the Lenses dimension in the discussion.

#### EBMgt concept

We identified several conceptual articles discussing EBMgt in healthcare ( Supplementary File 7 ) that did not fit the Model. Some of these articles discussed the main principles of EBMgt ( Axelsson, 1998 ). Others argued for its application to different healthcare management subfields, like healthcare human resource management ( Cohen, 2011 ). Finally, Hewison (2004) critiqued EBMgt, arguing that it is incongruent with current management practice.

## Discussion

The COVID-19 pandemic highlighted the necessity of using evidence in decision-making and put EBMgt at the forefront of hospital management. To better understand EBMgt in hospital settings and how to leverage it in practice, we conducted a scoping review, integrating current knowledge, identifying the gaps and delineating areas for future research. We developed a novel review methodology, which involved systematically searching the literature twice; first using EBMgt terminology, and second using terminology associated with the EBMgt concept. We integrated the resulting 218 articles into the Model and found that most of the articles could be captured by the model's dimensions, except for a handful of articles that discussed the EBMgt concept.

We make two major contributions to the literature. First, we identified the gaps in our knowledge of the EBMgt process in hospital settings and delineated areas for future research. The major gaps related to the lenses that influence the EBMgt process, the outcomes of EBMgt application and the representation of Global South countries in the English language literature. Second, we developed a new methodology of identifying keywords for scoping reviews that could capture the fragmented literature on EBMgt. Using this methodology, we not only gained a deeper understanding of the state of the knowledge on EBMgt, but also contribute a methodology that has promise for scoping reviews of interdisciplinary topics. We discuss these contributions in detail below.

### Gaps in our knowledge and future research

The research on EBMgt in hospital settings has focused on two aspects, the EBMgt concept and EBMgt application. Research on the EBMgt concept included conceptual articles discussing EBMgt principles and advocating its application. While the general management EBMgt literature is dominated by such articles ( Rynes and Bartunek, 2017 ; Reay *et al.* , 2009 ), they were not prominent in the current review. Rather, we found that unlike the general management setting ( Rynes and Bartunek, 2017 ), the articles on EBMgt in hospital settings are primarily empirical in nature, examining EBMgt application in practice.

Mapping these articles about EBMgt application onto the Model ( Sahakian, 2020 ), we found several areas that future research can focus on. For example, within the process of evidence-based decision-making, the step of appraising the evidence has not received much research attention. This scarcity is at odds with the fundamental premise of EBMgt that the quality of decisions is likely to improve the more managers use reliable evidence. As another example, the decision criteria have received little research attention. The identification of these criteria is starting to build a case that in addition to evidence certain contextual factors are also considered during EBMgt ( Sahakian, 2020 ). Thus, more research is necessary to examine the influence of these criteria on EBMgt. The results of such research could also be used to reflect on the Model and refine the representation of decision criteria within it. In this discussion, we will focus in-depth on three areas we believe pose major gaps in our knowledge of EBMgt in hospital settings.

#### Lenses: managers' subjectivity shaping EBMgt

Lenses, which represent managers' perceptions of situations and motives that impact the EBMgt decision-making process, were neglected in the literature. The strategy process literature highlights the importance of considering the influence of perceptions ( Pettigrew, 1992 ; Pettigrew *et al.* , 2001 ). This literature stresses that process cannot be discussed without considering how the subjective interpretation of actors within a certain context can change such processes. This aspect has been overlooked in EBMgt, although implementation of EBMgt involves a dynamic process where agents continuously interpret information in light of their knowledge, aims and power ( Baba and HakemZadeh, 2012 ). Critics of EBMgt have argued that to better understand issues like power, politics and ethics, there has to be a greater study of how managers perceive situations and how this impacts EBMgt practice ( Morrell and Learmonth, 2015 ). Moreover, the strategy process literature notes that actors use aspects of context to obtain outcomes important to them. Recent research into EBMgt has found preliminary evidence that managers use evidence for different purposes, including problem solving or giving legitimacy to predetermined actions ( Sahakian, 2020 ). Therefore, more research into the impact of managers' motives for using evidence is necessary to better understand EBMgt practice. This could possibly be done through using the critical incident technique to explore managers' motives and perceptions in specific incidents of EBMgt. It could also be done through using multiple case studies to examine different evidence-based decisions and managers' differing perceptions and motives. The results of such research could also be used to reflect on the Model and refine the representation of lenses within it.

#### EBMgt outcomes: evidence for effectiveness

We can view outcomes of EBMgt decision-making from a temporal perspective, in proximal and distal terms. Proximal outcomes are the outcomes targeted by a specific EBMgt decision. Distal outcomes refer to the long-term outcomes of EBMgt application that result from many factors. Our review includes many studies that showed that hospitals implementing evidence-based solutions attained positive outcomes, and better outcomes than before. To illustrate, Plantier *et al.* (2017) found a significant positive impact of implementing EHR on the hospital quality of care. Such studies presenting cases of EBMgt application provide evidence that EBMgt can improve proximal outcomes of a decision.

Our review did not reveal, however, any studies assessing the effectiveness of EBMgt as an overall decision-making approach. There is no evidence that hospitals that regularly apply EBMgt have better outcomes than before, or in comparison with hospitals that do not. Thus, evidence of distal outcomes of EBMgt application is lacking in the literature in hospital settings, similar to the EBMgt literature overall ( Rynes and Bartunek, 2017 ) and is an important area for future research. While methodologically challenging, longitudinal studies could be conducted with managers receiving EBMgt training that assess the extent of their implementation of EBMgt and its effect on different outcomes ( Rynes and Bartunek, 2017 ) . Alternatively, organizational outcomes can be examined before and after the implementation of certain organizational-level EBMgt facilitators.

#### The Global South: neglected in English language publications

Reflecting on the generalizability of the literature across contexts, we notice that certain national contexts were neglected in the literature. We found that most studies in this review were conducted in Global North countries. Similar to the literature on human behavior overall ( Thalmayer *et al.* , 2020 ), the English language literature on EBMgt in hospital settings has neglected about 90% of the World population, with only 15% of the articles representing Global South countries. This lack of representation may not be an omission of the literature but a limitation of our search, which involved only English language publications. However, we argue that the neglect of the Global South in the *English language* EBMgt literature is nonetheless a major gap.

English is the lingua franca of international science ( Di Bitetti and Ferreras, 2017 ), and it dominates much of the world's healthcare information ( Adams and Fleck, 2015 ). Furthermore, most of the journals included in prestigious journal indices (e.g. Web of Science, Scopus), which have broad readership and are where scientists try to submit their best work, are in English ( Mongeon and Paul-Hus, 2014 ). The lack of representation of the Global South in such publication is problematic because they starkly differ from the Global North in critical areas including income, education and health ( Henrich *et al.* , 2010 ). Given that most of our knowledge and understanding of EBMgt in hospital settings published in the English language is based on the Global North, this could have implications for the conclusion we can draw about EBMgt in differing national contexts and the theories we can build.

Additionally, while language of such publications may be a barrier, the more prominent issue is the generalizability of the findings to hospitals in the Global South, where the socio-politico-economic contexts, and consequently the healthcare systems, are very different. Whilst healthcare systems around the globe face challenges, there is a disparity between and within these systems in the Global North and South. Examples of these disparities relate to state capacity in terms of regulation and financing of the systems, burden of diseases, per-capita spending and the healthcare provider-inhabitant ratio ( de Carvalho *et al.* , 2021 ). Discrepancies at the system level therefore create healthcare organizational contexts where findings from studies dominating the literature may not be generalizable.

While this literature fit well under the Model, which is based on the Lebanese context, there were certain discrepancies. Certain sub-themes under barriers and facilitators (e.g. establishing national reforms) and decision criteria (e.g. impact of marketing initiatives) were unique to the literature, while others (e.g. technology) remained unique to the model. Thus, future English language research studying EBMgt in hospitals should focus on the remaining parts of the World to better understand the impact of differences in contexts.

### Methodological contribution: systematically scoping a fragmented literature

Systematically reviewing the EBMgt literature in hospital settings has a few challenges. Namely, the EBMgt terminology is new and not widespread ( Briner *et al.* , 2009 ), and research on EBMgt in healthcare management is spread across different disciplines, including management, medicine and nursing. To overcome these challenges, we developed a novel four-step methodology. While step one of our process; systematically searching the literature using EBMgt terminology, was not novel, the remaining steps were. These involved using an expert panel to identify the relevant keywords used by articles resulting from step one, conducting a second search using these keywords and reconciling the results across the searches. Using this methodology, we widened the scope of our search exponentially, identifying an additional 180 unique articles that did not use the EBMgt terminology, and better captured a fragmented literature that is dispersed across disciplines. As a result, our review provides a more holistic understanding of the current state of the knowledge on EBMgt in hospital settings.

Moreover, given the complexity of problems facing society today, great emphasis is placed on interdisciplinary research ( Pedersen, 2016 ). Our novel methodology can be used to capture all relevant terminology related to an interdisciplinary topic across different fields. It can therefore prove useful for synthesizing topics that, similar to EBMgt, are dispersed across different bodies of literature.

### Practical implications

This review provides managers some resources to facilitate EBMgt practice, including for example articles that discuss tools to support evidence acquisition, evaluation and use ( Lohr, 2004 ; Kibbe *et al.* , 1997 ). The review also indicates the different competencies that might be necessary for EBMgt; organizations could rely on these competencies to make selection decisions or to develop training programs. They can also use the barriers and facilitators to identify the role their internal structure, culture and practices play to support EBMgt, and to identify potential solutions. Researchers, educational institutions and government agencies can also use the barriers and facilitators to examine their role in hindering EBMgt practice, and the proposed solutions to take a more active role in enabling EBMgt application in practice.

### Limitations

Some limitations must be noted when considering the results of this study. First, we did not include the grey literature, because our study was the first scoping review of the literature on EBMgt using a new methodology. This may have limited our findings because grey literature can identify studies commissioned by organizations ( Briner *et al.* , 2009 ) and studies with null results ( Adams *et al.* , 2017 ) and provides an opportunity for future research. Second, not limiting the search to English language publications might have provided a better idea of the geographic distribution of the literature and provides an opportunity for future research. Third, our novel methodology, while exhaustive, was very time consuming. This explains why the latest studies in this review are two years old. This presents an opportunity to further refine this methodology.

## Conclusion

We set out to integrate the literature on EBMgt in hospital settings, identify the gaps and delineate areas for future research. We conducted a systematic scoping review, identified 128 articles, and categorized them within an EBMgt framework. We made two major contributions to the literature. First, we identified the major gaps in the literature on EBMgt in hospital settings and outlined areas for future research. We found that the English language literature provides limited insight into the role of managers' perceptions and motives in EBMgt, the practice of EBMgt in Global South countries, and the outcomes and effectiveness of EBMgt. Second, we developed a novel review methodology for reviewing phenomena, like EBMgt, that are not unified by common terminology and are studied across disciplines, thus contributing to the scoping review literature.

## Figures and Tables

**Table 1:** Juxtaposing three frameworks of EBMgt in healthcare

EBMgt framework	Assumption	Decision-making process		Considers context	Basis for development
		Sequential or dynamic	Single or Multi-level		
Kovner and Rundall (2006)	Rational decision-making	Sequential process	Single-level	No	Conceptual
Baba and HakemZadeh (2012)	Bounded rationality	Dynamic process	Multi-level	Yes	Conceptual
Sahakian (2020)	Bounded rationality	Sequential and iterative process	Multi-level	Yes	Empirical

**Table 2:** Data collection method of empirical articles

	Frequency	Percent
Single case study	58	38.67
Interview	47	31.33
Cross-sectional survey	38	25.33
Pretest-post-test design	18	12.00
Secondary data	9	6.00
Multiple case studies	7	4.67
Focus group discussion	4	2.67
Quasi-experimental design	4	2.67
Experimental design	2	1.33
Longitudinal design	2	1.33
Delphi study	1	0.67

## Appendix

The supplementary files are available online for this article.
