# Supplementary material for: The neglected contexts and outcomes of evidence-based management: a systematic scoping review in hospital settings
Source: J Health Organ Manag. 2021 Dec 28;36(9):48–65. doi: 10.1108/JHOM-03-2021-0101 (PMC9627724; doi:10.1108/JHOM-03-2021-0101)
Supplement: Supplementary file 1 [file JHOM-03-2021-0101_suppl1.docx]

**Supplemental File 1. Detailed Search and Filtering Strategy and Analysis**

We conducted a scoping review for peer-reviewed journal articles on the topic of EBMgt in hospital settings. We searched four online databases: PubMed, CINAHL, PsycINFO, and Cochrane Library, with no restrictions on publication year. These databases were selected to be wide in scope, encompassing disciplines related to health, life, social, and behavioral sciences, and business, to cover the hospital setting, and to be inclusive of all management levels.

The main challenges were that the EBMgt literature is spread across several fields and may not be using the EBMgt terminology. Existing reviews of the EBMgt literature have searched the literature either only using EBMgt terminology (i.e. EBMgt, evidence-informed management, Jaana *et al.*, 2014, Roshanghalb *et al.*, 2018, Rynes and Bartunek, 2017) or EBMgt terminology and related terms identified by expert librarians (i.e. research utilization, research to practice etc, Currie, 2013, Reay *et al.*, 2009).We adapted these two approaches and developed a novel methodology that involved two systematic searches; one using EBMgt terminology, and a second using terminology associated with the EBMgt concept, which we derived from the results of the first search. This resulted in a 4-step process, discussed further below, which we implemented from April 2015- October 2019. The novelty of this process is in its use of the results of a first search to derive more keywords to conduct a second more expanded search.

***General systematic search***

First, we searched the four databases using the key-terms: ‘evidence’ AND ‘based’ OR ‘driven’ OR ‘informed’ AND ‘healthcare’ AND ‘management’. We specified peer reviewed, English-only journal articles, involving only human subjects. We exported the results from the databases into Microsoft Excel and merged all the results to identify and remove duplicates. We applied four filters to narrow down the articles:

1. Removed titles that reflect clinical topics (e.g., alginate dressings for venous leg ulcers)
2. Removed titles that are irrelevant (e.g., comment on World War II repression)
3. Read abstracts keeping those related to non-clinical evidence-based decision-making, decision-making in healthcare, decision-making by healthcare managers, and practice guideline development
4. Read full texts keeping those related to evidence-based hospital processes, procedures, and design, non-clinical evidence-based decision-making by hospital managers, evidence-based decision-making in hospitals.

Two researchers, including one of the authors, applied each of the filters separately and then reconciled their differences after each filter, consulting the rest of the authors when necessary. The two researchers also reviewed the reference lists of the remaining relevant articles.

***Keyword identification***

Second, we extracted the keywords of the articles remaining from the first step to determine EBMgt related terminology that might be used by relevant articles. We developed an expert panel involving the researchers who applied the filters and two of the remaining authors. To ensure that the search would not be limited in scope, the panel removed keywords that pertain to specific fields (e.g. community-health), practices (e.g. telehomecare), and countries (e.g. Canada). They then reviewed the relevance of the remaining words to EBMgt and based on consensus decided on a list of 21 keywords (Table I).

| **Table I. Keywords Identified from the Focused Keyword Identification Step.** | |
| --- | --- |
| **Focused Keywords** | |
| decision makers | knowledge based value creation |
| decision making | organizational decision making |
| decision science | research capacity building |
| evidence | research transfer |
| evidence informed improvement | research use |
| evidence based design | research practice gap |
| evidence based management | scientific evidence |
| evidence based practice | strength of evidence |
| knowledge flow | translation* |
| knowledge management | knowledge transfer |
| knowledge process |  |
| *** Referring to transfer of knowledge or evidence into practice | |

***Keyword systematic search***

Third, we searched the four databases using the 21 keywords, in addition to “healthcare AND management NOT clinical”. We applied the same specifications as the first search, exported and merged the results, removed duplicates, and applied the same four filters.

***Reconciliation***

Fourth, to ensure the uniqueness of the final list of relevant articles from the two searches, we examined the overlap of the articles across the two searches and removed duplicates.

**Analysis**

We analyzed the articles using a deductive content analysis approach (Elo and Kyngas, 2008). The foundation for the categorization was the Grounded Model of the Evidence-based Management Process (hereafter the Model) with its five dimensions encompassing 30 themes.

***Coding***

We tabulated key information about each article including their objectives, methodology, results, and limitations on Microsoft Excel. Two of the authors conducted the categorization simultaneously and collaboratively. They familiarized themselves with each of the articles, paying attention to the objectives and results of each, and assigned each article a code based on the dimensions and themes of the Model. Articles were assigned to a relevant dimension or set of dimensions, as well as, theme or set of themes from the model. When articles did not fit the dimensions and themes provided by the model, we created new themes derived from the data to encompass these articles based on the principles of inductive content analysis (Elo and Kyngas, 2008).

***Reliability***

To increase reliability of the categorization of articles according to the Model we engaged an independent coder, a PhD candidate in organizational psychology. We provided the coder with definitions of each of the dimensions and, based on Lacy and Riffe’s (1996) recommendations, asked them to code a random sample of 70 articles. We assessed inter-coder reliability and found moderate agreement between our categorization and the independent coder’s, Cohen’s *κ* = .59 (95% CI, 0.44 to 0.75). We revisited the disagreements, made a change to the categorization of one article, and found that most disagreements were due to the coders’ lack of familiarity with the concepts.

**References**

Currie, K. M. (2013), "Updating Reay, Berta & Kohn EBMgt systematic review", University of Prince Edward Island, Canada.

Elo, S. and Kyngas, H. (2008), "The qualitative content analysis process.", *Journal of Advanced Nursing,* Vol. 62 No. 1, pp. 107-115.

Jaana, M., Vartak, S. and Ward, M. M. (2014), "Evidence-based health care management: what is the research evidence available for health care managers?", *Evaluation & the health professions,* Vol. 37 No. 3, pp. 314-334.

Lacy, S. and Riffe, D. (1996), " Sampling error and selecting intercoder reliability samples for nominal content categories.", *Journalism & Mass Communication Quarterly,* Vol. 73 No. 4, pp. 963-973.

Reay, T., Berta, W. and Kohn, M. K. (2009), "What's the evidence on evidence-based management?", *The Academy of Management Perspectives,* Vol. 23 No. 4, pp. 5-18.

Roshanghalb, A., Lettieri, E., Aloini, D., Cannavacciuolo, L., Gitto, S. and Visintin, F. (2018), "What evidence on evidence-based management in healthcare?", *Management Decision,* Vol. 56 No. 10, p. 2069.

Rynes, S. L. and Bartunek, J. M. (2017), "Evidence-Based Management: Foundations, Development, Controversies and Future", *Annual Review of Organizational Psychology and Organizational Behavior,* Vol. 4, pp. 235-261.
