# Supplementary material for: The neglected contexts and outcomes of evidence-based management: a systematic scoping review in hospital settings
Source: J Health Organ Manag. 2021 Dec 28;36(9):48–65. doi: 10.1108/JHOM-03-2021-0101 (PMC9627724; doi:10.1108/JHOM-03-2021-0101)
Supplement: Supplementary file 2 [file JHOM-03-2021-0101_suppl2.docx]

**Supplemental File 2. List of Articles Resulting from the Systematic Scoping Review**

Abidi, S. S. R. (1999), "Applying data mining in healthcare: an info-structure for delivering" data-driven" strategic services", *Studies in Health Technology and Informatics*, pp. 453-456.

AbuKhousa, E., Al-Jaroodi, J., Lazarova-Molnar, S. and Mohamed, N. H. (2014), "Simulation and modeling efforts to support decision making in healthcare supply chain management", *The Scientific World Journal,* Vol. 2014, pp. 1-16.

Adams, R., Rush, K., Leddy, C., Cook, T. L., Leach, T. K., Bollinger, B. I. and Hoyle, L. (2016), "Increased consumer communication and knowledge leads to higher quality in health care", *Radiologic Technology,* Vol. 88 No. 1, pp. 80-83.

Afilal, M., Yalaoui, F., Dugardin, F., Amodeo, L., Laplanche, D. and Blua, P. (2016), "Forecasting the emergency department patients flow", *Journal of Medical Systems,* Vol. 40 No. 7, p. 175.

Aguado-Correa, F., Herrera-Carranza, M. and Padilla-Garrido, N. (2016), "Variability and overcrowding management: ongoing challenge for spanish hospital emergency departments", *Journal of Health Management,* Vol. 18 No. 2, pp. 218-230.

Al-Hyari, K., Abu Hammour, S., Abu Zaid, M. K. S. and Haffar, M. (2016), "The impact of Lean bundles on hospital performance: does size matter?", *International Journal of Health Care Quality Assurance,* Vol. 29 No. 8, pp. 877-894.

Aldrich, R., Bonevski, B. and Wilson, A. (2006), "A case study on determining and responding to health managers' priorities for research to assist health service decision making", *Australian Health Review,* Vol. 30 No. 4, pp. 435-441.

Alexander, J. A., Hearld, L. R., Jiang, H. J. and Fraser, I. (2007), "Increasing the relevance of research to health care managers: hospital CEO imperatives for improving quality and lowering costs", *Health Care Management Review,* Vol. 32 No. 2, pp. 150-159.

Allen, J. (1997), "Overcome info overload to improve decision-making. ", *Data Strategy & Benchmarking,* Vol. 1 No. 6, pp. 81-96.

Astley, J. and Wake-Dyster, W. (2001), "Evidence-based priority setting", *Australian Health Review,* Vol. 24 No. 2, pp. 32-39.

Atack, L., Gignac, P. and Anderson, M. (2010), "Getting the right information to the table: using technology to support evidence-based decision making", *Healthcare Management Forum,* Vol. 23 No. 4, pp. 164-168.

Attree, M. (2001), "A study of the criteria used by healthcare professionals, managers and patients to represent and evaluate quality care", *Journal of Nursing Management,* Vol. 9 No. 2, pp. 67-78.

Axelsson, R. (1998), "Towards an evidence based health care management", *The International Journal of Health Planning and Management,* Vol. 13 No. 4, pp. 307-317.

Baghbanian, A., Hughes, I., Kebriaei, A. and Khavarpour, F. A. (2012), "Adaptive decision-making: how Australian healthcare managers decide", *Australian Health Review,* Vol. 36 No. 1, pp. 49-56.

Bai, J., Fügener, A., Schoenfelder, J. and Brunner, J. O. (2018), "Operations research in intensive care unit management: a literature review", *Health Care Management Science,* Vol. 21 No. 1, pp. 1-24.

Barak-Corren, Y., Israelit, S. H. and Reis, B. Y. (2017), "Progressive prediction of hospitalisation in the emergency department: uncovering hidden patterns to improve patient flow", *Emerg Med J,* Vol. 34 No. 5, pp. 308-314.

Barton, A. J. (1994), "Data needs for decision support of chief nurse executives", *The Journal of Nursing Administration,* Vol. 24 No. 4 Suppl, pp. 19-25.

Beglinger, J. E. (2006), "Quantifying patient care intensity: an evidence-based approach to determining staffing requirements", *Nursing Administration Quarterly,* Vol. 30 No. 3, pp. 193-202.

Bell, A. M., Bohannon, J., Porthouse, L., Thompson, H. and Vago, T. (2016), "Process improvement to enhance quality in a large volume labor and birth unit", *MCN: The American Journal of Maternal/Child Nursing,* Vol. 41 No. 6, pp. 340-348.

Bellamkonda, V. R., Kumar, R., Scanlan-Hanson, L. N., Hess, J. J., Hellmich, T. R., Bellamkonda, E., Campbell, R. L., Hess, E. P. and Nestler, D. M. (2016), "Pilot study of Kano “Attractive Quality” techniques to identify change in emergency department patient experience", *Annals of Emergency Medicine,* Vol. 68 No. 5, pp. 553-561.

Bendesky, B. S., Hunter, K., Kirchhoff, M. A. and Jones, C. W. (2016), "Same physician, different location, different patient satisfaction scores", *Annals of Emergency Medicine,* Vol. 68 No. 5, pp. 531-535.

Berry, L. L., Parker, D., Coile, R. C., Hamilton, D. K., O Neill, D. D. and Sadler, B. L. (2004), "The business case for better buildings", *Frontiers of Health Services Management,* Vol. 21, pp. 3-24.

Bigelow, B. and Arndt, M. (2003), "Teaching evidence-based management: where do we go from here?", *The Journal of Health Administration Education,* Vol. 20 No. 4, pp. 305-312.

Bowen, M., Prater, A., Safdar, N. M., Dehkharghani, S. and Fountain, J. A. (2016), "Utilization of workflow process maps to analyze gaps in critical event notification at a large, urban hospital", *Journal of Digital Imaging,* Vol. 29 No. 4, pp. 420-424.

Brady, A.-M., Byrne, G., Quirke, M. B., Lynch, A., Ennis, S., Bhangu, J. and Prendergast, M. (2017), "Barriers to effective, safe communication and workflow between nurses and non-consultant hospital doctors during out-of-hours", *International Journal for Quality in Health Care,* Vol. 29 No. 7, pp. 929-934.

Browman, G. P., Snider, A. and Ellis, P. (2003), "Negotiating for change. The healthcare manager as catalyst for evidence-based practice: changing the healthcare environment and sharing experience", *HealthcarePapers,* Vol. 3 No. 3, pp. 10-22.

Brown, C. E. and Ecoff, L. (2011), "A systematic approach to the inclusion of evidence in healthcare design", *HERD: Health Environments Research & Design Journal,* Vol. 4 No. 2, pp. 7-16.

Bucci, S., De Belvis, A., Marventano, S., De Leva, A., Tanzariello, M., Specchia, M. L., Ricciardi, W. and Franceschi, F. (2016), "Emergency Department crowding and hospital bed shortage: is Lean a smart answer? A systematic review", *Eur Rev Med Pharmacol Sci,* Vol. 20 No. 20, pp. 4209-19.

Büchner, V. A., Hinz, V. and Schreyögg, J. (2016), "Health systems: changes in hospital efficiency and profitability", *Health Care Management Science,* Vol. 19 No. 2, pp. 130-143.

Burgess, N. and Currie, G. (2013), "The knowledge brokering role of the hybrid middle level manager: The case of healthcare", *British Journal of Management,* Vol. 24, pp. S132-S142.

Butler, V., Clinton, C., Sagi, H. K., Kenney, R. and Barsoum, W. K. (2012), "Applying science and strategy to operating room workforce management", *Nursing Economics,* Vol. 30 No. 5, p. 275.

Buttigieg, S. C., Gauci, D. and Dey, P. (2016), "Continuous quality improvement in a Maltese hospital using logical framework analysis", *Journal of Health Organization and Management,* Vol. 30 No. 7, pp. 1026-1046.

Calegari, R., Fogliatto, F. S., Lucini, F. R., Neyeloff, J., Kuchenbecker, R. S. and Schaan, B. D. (2016), "Forecasting daily volume and acuity of patients in the emergency department", *Computational and Mathematical Methods in Medicine,* Vol. 2016, pp. 1-8.

Canaway, R., Bismark, M., Dunt, D. and Kelaher, M. (2017), "Medical directors’ perspectives on strengthening hospital quality and safety", *Journal of Health Organization and Management,* Vol. 31 No. 7/8, pp. 696-712.

Capan, M., Khojandi, A., Denton, B. T., Williams, K. D., Ayer, T., Chhatwal, J., Kurt, M., Lobo, J. M., Roberts, M. S. and Zaric, G. (2017), "From data to improved decisions: Operations Research in healthcare delivery", *Medical Decision Making,* Vol. 37 No. 8, pp. 849-859.

Card, A. J., Ward, J. R. and Clarkson, P. J. (2012), "Getting to Zero: Evidence‐based healthcare risk management is key", *Journal of Healthcare Risk Management,* Vol. 32 No. 2, pp. 20-27.

Carnero, M. C. and Gómez, A. (2016), "A multicriteria decision making approach applied to improving maintenance policies in healthcare organizations", *BMC Medical Informatics and Decision Making,* Vol. 16 No. 1, p. 47.

Champagne, F., Lemieux-Charles, L., Duranceau, M.-F., MacKean, G. and Reay, T. (2014), "Organizational impact of evidence-informed decision making training initiatives: a case study comparison of two approaches", *Implementation Science,* Vol. 9 No. 1, p. 53.

Chan, K. S., Morton, S. C. and Shekelle, P. G. (2004), "Systematic reviews for evidence-based management: how to find them and what to do with them", *American Journal of Managed Care,* Vol. 10 No. 11 Pt 1, pp. 806-12.

Chen, K.-C., Chien, L.-N., Hsu, Y.-H. and Yu, M.-M. (2016), "Metafrontier frameworks for studying hospital productivity growth and quality changes", *International Journal for Quality in Health Care,* Vol. 28 No. 6, pp. 650-656.

Chiarini, A. and Baccarani, C. (2016), "TQM and lean strategy deployment in Italian hospitals: Benefits related to patient satisfaction and encountered pitfalls", *Leadership in health services,* Vol. 29 No. 4, pp. 377-391.

Chow, C. W., Ganulin, D., Haddad, K. and Harrison, P. D. (1999), "Increasing the effectiveness of resource deployment in healthcare organizations", *Journal of Healthcare Management,* Vol. 44 No. 6, pp. 513-528.

Clancy, C. M. and Cronin, K. (2005), "Evidence-based decision making: global evidence, local decisions", *Health Affairs,* Vol. 24 No. 1, pp. 151-162.

Claret, P. G., Bobbia, X., Olive, S., Demattei, C., Yan, J., Cohendy, R., Landais, P. and de La Coussaye, J. E. (2016), "The impact of emergency department segmentation and nursing staffing increase on inpatient mortality and management times", *BMC Health Services Research,* Vol. 16 No. 1, p. 279.

Cohen, K. R. (2011), "The case for evidence-based human capital management.", *Healthcare Financial Management,* Vol. 65 No. 8, pp. 102-108.

da Silveira Grübler, M., da Costa, C. A., da Rosa Righi, R., Rigo, S. J. and Chiwiacowsky, L. D. (2018), "A Hospital bed allocation hybrid model based on situation awareness", *CIN: Computers, Informatics, Nursing,* Vol. 36 No. 5, pp. 249-255.

Davidson, J. E. (2017), "Organizing the evidence for healthcare design projects", *HERD: Health Environments Research & Design Journal,* Vol. 10 No. 2, pp. 13-22.

de-Carvalho, D., Alvim-Borges, J. L. and Toscano, C. M. (2017), "Impact assessment of an automated drug-dispensing system in a tertiary hospital", *Clinics,* Vol. 72 No. 10, pp. 629-636.

Debono, D., Taylor, N., Lipworth, W., Greenfield, D., Travaglia, J., Black, D. and Braithwaite, J. (2017), "Applying the theoretical domains framework to identify barriers and targeted interventions to enhance nurses’ use of electronic medication management systems in two Australian hospitals", *Implementation Science,* Vol. 12 No. 1, p. 42.

Delias, P., Doumpos, M., Grigoroudis, E., Manolitzas, P. and Matsatsinis, N. (2015), "Supporting healthcare management decisions via robust clustering of event logs", *Knowledge-Based Systems,* Vol. 84, pp. 203-213.

DeRienzo, C. M., Shaw, R. J., Meanor, P., Lada, E., Ferranti, J. and Tanaka, D. (2017), "A discrete event simulation tool to support and predict hospital and clinic staffing", *Health Informatics Journal,* Vol. 23 No. 2, pp. 124-133.

Devine, K., Ealey, T. and O'Clock, P. (2008), "A framework for cost management and decision support across health care organizations of varying size and scope", *Journal of Health Care Finance,* Vol. 35 No. 2, pp. 63-75.

DeWulf, A., Otchi, E. H. and Soghoian, S. (2017), "Identifying priorities for quality improvement at an emergency Department in Ghana", *BMC Emergency Medicine,* Vol. 17 No. 1, p. 28.

Donetto, S., Penfold, C., Anderson, J., Robert, G. and Maben, J. (2017), "Nursing work and sensory experiences of hospital design: a before and after qualitative study following a move to all-single room inpatient accommodation", *Health & Place,* Vol. 46, pp. 121-129.

Doods, S. (2005), "Designing improved health care processes using discrete event stimulation", *Br J Healthcare Comput Inf Manag,* Vol. 22, pp. 14-16.

Doorduijn, A. S., van Gameren, Y., Vasse, E. and de Roos, N. M. (2016), "At Your Request® room service dining improves patient satisfaction, maintains nutritional status, and offers opportunities to improve intake", *Clinical Nutrition,* Vol. 35 No. 5, pp. 1174-1180.

Eiset, A. H., Erlandsen, M., Møllekær, A. B., Mackenhauer, J. and Kirkegaard, H. (2016), "A generic method for evaluating crowding in the emergency department", *BMC Emergency Medicine,* Vol. 16 No. 1, p. 21.

Elamir, H. (2018), "Improving patient flow through applying lean concepts to emergency department", *Leadership in Health Services,* Vol. 31 No. 3, pp. 293-309.

Ellen, M. E., Lavis, J. N., Ouimet, M., Grimshaw, J. M. and Bédard, P. O. (2011), "Determining research knowledge infrastructure for healthcare systems: a qualitative study", *Implementation Science,* Vol. 6 No. 1, p. 60.

Ellen, M. E., Léon, G., Bouchard, G., Lavis, J. N., Ouimet, M. and Grimshaw, J. M. (2013), "What supports do health system organizations have in place to facilitate evidence-informed decision-making?A qualitative study", *Implementation Science,* Vol. 8 No. 1, pp. 1-19.

Ellen, M. E., Léon, G., Bouchard, G., Ouimet, M., Grimshaw, J. M. and Lavis, J. N. (2014), "Barriers, facilitators and views about next steps to implementing supports for evidence-informed decision-making in health systems: a qualitative study", *Implementation Science,* Vol. 9 No. 1, p. 179.

Fagerström, L. (2009), "Evidence‐based human resource management: a study of nurse leaders' resource allocation", *Journal of Nursing Management,* Vol. 17 No. 4, pp. 415-425.

Fanelli, S. and Zangrandi, A. (2017), "Assessment for improving the performance of NICUs: The Italian experience", *Health Services Management Research,* Vol. 30 No. 3, pp. 168-178.

Ferlie, E. and Wood, M. (2003), "Novel mode of knowledge production? Producers and consumers in health services research", *Journal of health services research & policy,* Vol. 8 No. 2_suppl, pp. 51-57.

Fernandez, A., Schrogie, J., Wilson, W. and Nash, D. (1997), "Technology assessment in healthcare: a review and description of a" best practice" technology assessment process", *Best Practices and Benchmarking in Healthcare,* Vol. 2 No. 6, pp. 240-253.

Finkelstein, B. S., Silvers, J. and Rosenthal, G. E. (1997), "The importance of outcomes data in health care decision making and purchasing", *Marketing health services,* Vol. 17 No. 2, p. 52.

Finkler, S. A. (2002), "Teaching future healthcare financial managers to use evidence", *The Journal of Health Administration Education,* Vol. 20 No. 4, pp. 243-261.

Finkler, S. A. (2004), "Evidence-Based Financial Management - What are We Waiting for?", *Research in Healthcare Financial Management,* Vol. 9 No. 1, p. 1.

Finkler, S. A. and Ward, D. M. (2003), "The case for the use of evidence-based management research for the control of hospital costs", *Health Care Management Review,* Vol. 28 No. 4, pp. 348-365.

Fischer, M. D., Dopson, S., Fitzgerald, L., Bennett, C., Ferlie, E., Ledger, J. and McGivern, G. (2016), "Knowledge leadership: Mobilizing management research by becoming the knowledge object", *Human Relations,* Vol. 69 No. 7, pp. 1563-1585.

Fletcher, L. and Thornhill, J. (2009), "Organizational value in enhancing individual research use capacity: a joint evaluation project led by EXTRA and SEARCH Canada", *Healthcare Quarterly,* Vol. 12 No. 2, pp. 18-20.

Foglia, E., Lettieri, E., Ferrario, L., Porazzi, E., Garagiola, E., Pagani, R., Bonfanti, M., Lazzarotti, V., Manzini, R. and Masella, C. (2017), "Technology assessment in hospitals: lessons learned from an empirical experiment", *International Journal of Technology Assessment in Health Care,* Vol. 33 No. 2, pp. 288-296.

Friedman, C. P. (1999), "Information technology leadership in academic medical centers: a tale of four cultures", *Academic Medicine,* Vol. 74 No. 7, pp. 795-799.

Fulbrook, P., Jessup, M. and Kinnear, F. (2017), "Implementation and evaluation of a ‘Navigator’role to improve emergency department throughput", *Australasian Emergency Nursing Journal,* Vol. 20 No. 3, pp. 114-121.

Gagliardi, A. R. and Dobrow, M. J. (2016), "Identifying the conditions needed for integrated knowledge translation (IKT) in health care organizations: qualitative interviews with researchers and research users", *BMC Health Services Research,* Vol. 16 No. 1, p. 256.

Gallego, G., Fowler, S. and van Gool, K. (2008), "Decision makers' perceptions of health technology decision making and priority setting at the institutional level", *Australian Health Review,* Vol. 32 No. 3, pp. 520-527.

Gartnera, D. and Padmanb, R. (2017), "E-HOSPITAL–a digital workbench for hospital operations and services planning using information technology and algebraic languages", *Studies in Health Technology and Informatics,* Vol. 245, pp. 84-88.

Gautam, K. (2008), "Addressing the research-practice gap in healthcare management", *Journal of Public Health Management and Practice,* Vol. 14 No. 2, pp. 155-159.

Gignon, M., Amsallem, C. and Ammirati, C. (2017), "Moving a hospital: simulation–a way to co-produce safety healthcare facilities", *International Journal of Occupational Safety and Ergonomics,* Vol. 23 No. 4, pp. 589-591.

Gillespie, A. and Reader, T. W. (2016), "The Healthcare Complaints Analysis Tool: development and reliability testing of a method for service monitoring and organisational learning", *BMJ Qual Saf,* Vol. 25 No. 12, pp. 937-946.

Ginsburg, L. S. (2003), "Factors that influence line managers' perceptions of hospital performance data", *Health Services Research,* Vol. 38 No. 1p1, pp. 261-286.

Gold, B., England, D., Riley, W., Jacobs-Halsey, G., Webb, C. and Daniels, B. (2016), "Integrating quality improvement and continuing professional development at an academic medical center: a partnership between practice plan, hospital, and medical school", *Journal of Continuing Education in the Health Professions,* Vol. 36 No. 4, pp. 307-315.

Golenko, X., Pager, S. and Holden, L. (2012), "A thematic analysis of the role of the organisation in building allied health research capacity: a senior managers’ perspective", *BMC Health Services Research,* Vol. 12 No. 1, p. 276.

Green, A. (2011), "Information overload in healthcare management: How the READ Portal is helping healthcare managers", *Journal of the Canadian Health Libraries Association/Journal de l'Association des bibliothèques de la santé du Canada,* Vol. 32 No. 3, pp. 173-176.

Guo, R., Berkshire, S. D., Fulton, L. V. and Hermanson, P. M. (2017), "Use of evidence-based management in healthcare administration decision-making", *Leadership in Health Services,* Vol. 30 No. 3, pp. 330-342.

Guzman, G., Fitzgerald, J. A., Fulop, L., Hayes, K., Poropat, A., Avery, M., Campbell, S., Fisher, R., Gapp, R. and Herington, C. (2015), "How best practices are copied, transferred, or translated between health care facilities: a conceptual framework", *Health Care Management Review,* Vol. 40 No. 3, pp. 193-202.

Härkänen, M., Saano, S. and Vehviläinen‐Julkunen, K. (2017), "Using incident reports to inform the prevention of medication administration errors", *Journal of Clinical Nursing,* Vol. 26 No. 21-22, pp. 3486-3499.

Hawkins, J. B., Brownstein, J. S., Tuli, G., Runels, T., Broecker, K., Nsoesie, E. O., McIver, D. J., Rozenblum, R., Wright, A. and Bourgeois, F. T. (2016), "Measuring patient-perceived quality of care in US hospitals using Twitter", *BMJ Qual Saf,* Vol. 25 No. 6, pp. 404-413.

Hewison, A. (2004), "Evidence-based management in the NHS: is it possible?", *Journal of Health Organization and Management,* Vol. 18 No. 5, pp. 336-348.

Hicks, C. W., Liu, J., Yang, W. W., DiBrito, S. R., Johnson, D. J., Brito, A., Higgins, R. S., Frank, S. M. and Wick, E. C. (2017), "A comprehensive Choosing Wisely quality improvement initiative reduces unnecessary transfusions in an Academic Department of Surgery", *The American Journal of Surgery,* Vol. 214 No. 4, pp. 571-576.

Holland, D. E., Brandt, C., Targonski, P. V. and Bowles, K. H. (2017), "Validating performance of a hospital discharge planning decision tool in community hospitals", *Professional case management,* Vol. 22 No. 5, pp. 204-213.

Ibrahim, A. M., Dimick, J. B. and Joseph, A. (2017), "Building a better operating room: views from surgery and architecture", *Annals of Surgery,* Vol. 265 No. 1, pp. 34-36.

Inal, T. C., Goruroglu Ozturk, O., Kibar, F., Cetiner, S., Matyar, S., Daglioglu, G. and Yaman, A. (2018), "Lean six sigma methodologies improve clinical laboratory efficiency and reduce turnaround times", *Journal of Clinical Laboratory Analysis,* Vol. 32 No. 1, p. e22180.

Innis, J. and Berta, W. (2016), "Routines for change: how managers can use absorptive capacity to adopt and implement evidence‐based practice", *Journal of nursing management,* Vol. 24 No. 6, pp. 718-724.

Jan, S. (2003), "Why does economic analysis in health care not get implemented more? towards a greater understanding of the rules of the game and the costs of decision making", *Applied Health Economics and Health Policy,* Vol. 2 No. 1, pp. 17-24.

Janati, A., Hasanpoor, E., Hajebrahimi, S. and Sadeghi-Bazargani, H. (2018), "Evidence-based management–healthcare manager viewpoints", *International Journal of Health Care Quality Assurance,* Vol. 31 No. 5, pp. 436-448.

Jayakumar, K. L., Lavenberg, J. A., Mitchell, M. D., Doshi, J. A., Leas, B., Goldmann, D. R., Williams, K., Brennan, P. J. and Umscheid, C. A. (2016), "Evidence synthesis activities of a hospital evidence‐based practice center and impact on hospital decision making", *Journal of hospital medicine,* Vol. 11 No. 3, pp. 185-192.

Jbilou, J., Amara, N. and Landry, R. (2007), "Research based-decision-making in Canadian health organizations: a behavioural approach", *Journal of medical systems,* Vol. 31 No. 3, pp. 185-196.

Jbilou, J., Landry, R., Amara, N. and El Adlouni, S. (2009), "Combining communication technology utilization and organizational innovation: evidence from Canadian healthcare decision makers", *Journal of medical systems,* Vol. 33 No. 4, pp. 275-286.

Jessup, M., Crilly, J., Boyle, J., Wallis, M., Lind, J., Green, D. and Fitzgerald, G. (2016), "Users’ experiences of an emergency department patient admission predictive tool: A qualitative evaluation", *Health Informatics Journal,* Vol. 22 No. 3, pp. 618-632.

Jiang, S. and Verderber, S. (2017), "On the planning and design of hospital circulation zones: a review of the evidence-based literature", *HERD: Health Environments Research & Design Journal,* Vol. 10 No. 2, pp. 124-146.

Jih, W.-J., Chen, C.-H. and Chen, Y.-H. (2006), "Effects of knowledge management implementation in hospitals: An exploratory study in Taiwan", *International Journal of Knowledge Management (IJKM),* Vol. 2 No. 3, pp. 1-20.

Johnson, K., M. Mazur, L., Chadwick, J., Pooya, P., Amos, A. and McCreery, J. (2017), "Integrating lean exploration loops into healthcare facility design: Schematic phase", *HERD: Health Environments Research & Design Journal,* Vol. 10 No. 3, pp. 131-141.

Juzwishin, D. (2010), "Evidence informed decision-making in healthcare: the case for health technology assessment", *World hospitals and Health Services: The Official Journal of the International Hospital Federation,* Vol. 46 No. 1, pp. 10-12.

Karamitri, I., Talias, M. A. and Bellali, T. (2017), "Knowledge management practices in healthcare settings: a systematic review", *The International Journal of Health Planning and Management,* Vol. 32 No. 1, pp. 4-18.

Karliner, L. S., Pérez-Stable, E. J. and Gregorich, S. E. (2017), "Convenient access to professional interpreters in the hospital decreases readmission rates and estimated hospital expenditures for patients with limited English proficiency", *Medical care,* Vol. 55 No. 3, p. 199.

Khalifa, M. (2017), "Reducing length of stay by enhancing patients' discharge: a practical approach to improve hospital efficiency", *Studies in health technology and informatics,* Vol. 238, pp. 157-160.

Khalifa, M. and Zabani, I. (2016a), "Reducing emergency department crowding: evidence based strategies", *Studies in health technology and informatics,* Vol. 226, pp. 67-70.

Khalifa, M. and Zabani, I. (2016b), "Utilizing health analytics in improving the performance of healthcare services: a case study on a tertiary care hospital", *Journal of Infection and Public Health,* Vol. 9 No. 6, pp. 757-765.

Kibbe, D. C., Smith, P. P., LaVallee, R., Bailey, D. and Bard, M. (1997), "A guide to finding and evaluating best practices health care information on the Internet: the truth is out there?", *The Joint Commission Journal on Quality and Patient Safety,* Vol. 23 No. 12, pp. 678-689.

Kontio, E., Lundgren-Laine, H., Kontio, J., Korvenranta, H. and Salantera, S. (2013), "Information utilization in tactical decision making of middle management health managers", *Comput Inform Nurs,* Vol. 31 No. 1, pp. 9-16.

Korlén, S., Essén, A., Lindgren, P., Amer-Wahlin, I. and von Thiele Schwarz, U. (2017), "Managerial strategies to make incentives meaningful and motivating", *Journal of Health Organization and Management,* Vol. 31 No. 2, pp. 126-141.

Kovner, A. R., Elton, J. J. and Billings, J. (2000), "Evidence-based management / commentaries / reply", *Frontiers of Health Services Management,* Vol. 16 No. 4, pp. 3-46.

Kovner, A. R. and Rundall, T. G. (2006), "Evidence-based management reconsidered", *Frontiers of Health Services Management,* Vol. 22 No. 3, pp. 3-22.

Kovner, A. R., Wagner, R. F. and Curtis, R. S. (2001), "Better information for the board/Practitioner application", *Journal of Healthcare Management,* Vol. 46 No. 1, p. 53.

Kowalski, C., Yeaton, W. H., Kuhr, K. and Pfaff, H. (2017), "Helping hospitals improve patient centeredness: assessing the impact of feedback following a best practices workshop", *Evaluation & the Health Professions,* Vol. 40 No. 2, pp. 180-202.

Kreindler, S. A. (2008), "Watching your wait: evidence-informed strategies for reducing health care wait times", *Quality Management in Healthcare,* Vol. 17 No. 2, pp. 128-135.

Krugman, M., Sanders, C. and Kinney, L. J. (2015), "Part 2: Evaluation and outcomes of an evidence-based facility design project", *JONA: The Journal of Nursing Administration,* Vol. 45 No. 2, pp. 84-92.

Krugman, M. E. and Sanders, C. L. (2016), "Implementing a nurse manager profile to improve unit performance", *JONA: The Journal of Nursing Administration,* Vol. 46 No. 6, pp. 345-351.

Kullberg, A., Bergenmar, M. and Sharp, L. (2016), "Changed nursing scheduling for improved safety culture and working conditions–patients' and nurses' perspectives", *Journal of nursing management,* Vol. 24 No. 4, pp. 524-532.

Kyratsis, Y., Ahmad, R. and Holmes, A. (2012), "Making sense of evidence in management decisions: the role of research-based knowledge on innovation adoption and implementation in healthcare. Study protocol. 1.", *Implementation Science,* Vol. 7 No. 1, pp. 1-7.

Langaneer, J. R. I. and Worthington, D. (2010), "Operations research diffusion in health care management", *Journal of Health Care Finance,* Vol. 36 No. 3, pp. 73-87.

Lavoie‐Tremblay, M., Anderson, M., Bonneville‐Roussy, A., Drevniok, U. and Lavigne, G. L. (2012a), "Nurse executives’ perceptions of the executive training for research application (extra) program", *Worldviews on Evidence‐Based Nursing,* Vol. 9 No. 3, pp. 186-192.

Lavoie‐Tremblay, M., Richer, M. C., Marchionni, C., Cyr, G., Biron, A. D., Aubry, M., Bonneville‐Roussy, A. and Vézina, M. (2012b), "Implementation of evidence‐based practices in the context of a redevelopment project in a Canadian healthcare organization", *Journal of Nursing Scholarship,* Vol. 44 No. 4, pp. 418-427.

Leatherman, S. and Sutherland, K. (2007), "Designing national quality reforms: a framework for action", *International Journal for Quality in Health Care,* Vol. 19 No. 6, pp. 334-340.

Liang, Z., Howard, P. F., Leggat, S. G. and Murphy, G. (2012), "A framework to improve evidence-informed decision-making in health service management", *Australian Health Review,* Vol. 36 No. 3, pp. 284-289.

Lohr, K. N. (2004), "Rating the strength of scientific evidence: relevance for quality improvement programs", *International Journal for Quality in Health Care,* Vol. 16 No. 1, pp. 9-18.

Lomas, J. (2005), "Using research to inform healthcare managers’ and policy makers’ questions: from summative to interpretive synthesis", *Healthcare Policy,* Vol. 1 No. 1, p. 55.

Lovett, P. B., Illg, M. L. and Sweeney, B. E. (2016), "A successful model for a comprehensive patient flow management center at an academic health system", *American Journal of Medical Quality,* Vol. 31 No. 3, pp. 246-255.

Lucini, F. R., Fogliatto, F. S., da Silveira, G. J., Neyeloff, J. L., Anzanello, M. J., Kuchenbecker, R. d. S. and Schaan, B. D. (2017), "Text mining approach to predict hospital admissions using early medical records from the emergency department", *International Journal of Medical Informatics,* Vol. 100, pp. 1-8.

Luo, L., Luo, L., Zhang, X. and He, X. (2017), "Hospital daily outpatient visits forecasting using a combinatorial model based on ARIMA and SES models", *BMC Health Services Research,* Vol. 17 No. 1, p. 469.

Maass, K. L., Liu, B., Daskin, M. S., Duck, M., Wang, Z., Mwenesi, R. and Schapiro, H. (2017), "Incorporating nurse absenteeism into staffing with demand uncertainty", *Health Care Management Science,* Vol. 20 No. 1, pp. 141-155.

Mahmoudian-Dehkordi, A. and Sadat, S. (2017), "Sustaining critical care: using evidence-based simulation to evaluate ICU management policies", *Health Care Management Science,* Vol. 20 No. 4, pp. 532-547.

Margrif, F. D. (1991), "The role of health information managers in hospital financial management", *Journal of AHIMA,* Vol. 62 No. 11, pp. 59-61.

Marshall, M. N. (2013), "Bridging the ivory towers and the swampy lowlands; increasing the impact of health services research on quality improvement", *International Journal for Quality in Health Care,* Vol. 26 No. 1, pp. 1-5.

Matchar, D. B., Westermann-Clark, E. V., McCrory, D. C., Patwardhan, M., Samsa, G., Kulasingam, S., Myers, E., Sarria-Santamera, A., Lee, A. and Gray, R. (2005), "Dissemination of evidence-based practice center reports", *Annals of Internal Medicine,* Vol. 142 No. 12_Part_2, pp. 1120-1125.

Mathew, J. L. (2011), "KNOW ESSENTIALS: a tool for informed decisions in the absence of formal HTA systems", *International Journal of Technology Assessment in Health Care,* Vol. 27 No. 2, pp. 139-150.

Mazur, L. M., Johnson, K., Pooya, P., Chadwick, J. and McCreery, J. (2017), "Integrating lean exploration loops into healthcare facility design: programming phase", *HERD: Health Environments Research & Design Journal,* Vol. 10 No. 3, pp. 116-130.

Murphy, L. S., Wilson, M. L. and Newhouse, R. P. (2013), "Data analytics: making the most of input with strategic output", *JONA: The Journal of Nursing Administration,* Vol. 43 No. 7/8, pp. 367-370.

Myers, G., Côté‐Arsenault, D., Worral, P., Rolland, R., Deppoliti, D., Duxbury, E., Stoecker, M. and Sellers, K. (2016), "A cross‐hospital exploration of nurses’ experiences with horizontal violence", *Journal of nursing management,* Vol. 24 No. 5, pp. 624-633.

Mykkänen, M., Miettinen, M. and Saranto, K. (2016), "Standardized nursing documentation supports evidence-based nursing management", *Studies in health technology and informatics,* Vol. 225, pp. 466-470.

Naidoo, L. and Mahomed, O. H. (2016), "Impact of Lean on patient cycle and waiting times at a rural district hospital in KwaZulu-Natal", *African Journal of Primary Health Care & Family Medicine,* Vol. 8 No. 1, pp. 1084-1092.

Nantsupawat, A., Kunaviktikul, W., Nantsupawat, R., Wichaikhum, O. A., Thienthong, H. and Poghosyan, L. (2017), "Effects of nurse work environment on job dissatisfaction, burnout, intention to leave", *International Nursing Review,* Vol. 64 No. 1, pp. 91-98.

Nates, L. K. C., Pereira, A. J., Neto, A. C. and Silva, E. (2017), "A Quality initiative to implement a managed sepsis protocol in a public hospital based on the IHI quality improvement model: experience report", *Quality in Primary Care,* Vol. 25 No. 5, pp. 326-334.

Nelson, J. J. and Staffileno, B. A. (2017), "Improving the patient experience: call light intervention bundle", *Journal of pediatric nursing,* Vol. 36, pp. 37-43.

Newhouse, R. P. and White, K. M. (2011), "Guiding implementation: frameworks and resources for evidence translation", *JONA: The Journal of Nursing Administration,* Vol. 41 No. 12, pp. 513-516.

Nicklin, W. and Stipich, N. (2005), "Enhancing skills for evidence-based healthcare leadership: the Executive Training for Research Application (EXTRA) program", *Nursing Leadership,* Vol. 18 No. 3, pp. 35-44.

Niedzwiedzka, B. M. (2003), "Barriers to evidence-based decision making among Polish healthcare managers", *Health Services Management Research,* Vol. 16 No. 2, pp. 106-115.

Nilsson, K., Bååthe, F., Andersson, A. E., Wikström, E. and Sandoff, M. (2017), "Experiences from implementing value-based healthcare at a Swedish University Hospital–a longitudinal interview study", *BMC Health Services Research,* Vol. 17 No. 1, p. 169.

Nippak, P. M., Veracion, J. I., Muia, M., Ikeda-Douglas, C. J. and Isaac, W. W. (2016), "Designing and evaluating a balanced scorecard for a health information management department in a Canadian urban non-teaching hospital", *Health Informatics Journal,* Vol. 22 No. 2, pp. 120-139.

Novati, R., Papalia, R., Peano, L., Gorraz, A., Artuso, L., Canta, M., Del Vescovo, G. and Galotto, C. (2017), "Effectiveness of an hospital bed management model: results of four years of follow-up", *Annali di Igiene: Medicina Preventiva e di Comunita,* Vol. 29 No. 3, pp. 189-196.

Oetjen, R. M., Oetjen, D. M. and Rotarius, T. (2008), "Administrative decision making: a stepwise method", *The Health Care Manager,* Vol. 27 No. 1, pp. 4-12.

Ouimet, M., Lavis, J. N., Léon, G., Ellen, M. E., Bédard, P. O., Grimshaw, J. M. and Gagnon, M. P. (2014), "A cross-sectional survey of supports for evidence-informed decision-making in healthcare organisations: a research protocol", *Implementation Science,* Vol. 9 No. 1, p. 146.

Ovretveit, J. (1999), "Evaluation informed management and clinical governance", *British Journal of Clinical Governance,* Vol. 4 No. 3, pp. 103-109.

Ozyapici, H. and Tanis, V. N. (2016), "Improving health care costing with resource consumption accounting", *International Journal of Health Care Quality Assurance,* Vol. 29 No. 6, pp. 646-663.

Parente, C. A., Salvatore, D., Gallo, G. M. and Cipollini, F. (2018), "Using overbooking to manage no-shows in an Italian healthcare center", *BMC Health Services Research,* Vol. 18 No. 1, p. 185.

Patidar, N., Weech-Maldonado, R., O’Connor, S. J., Sen, B. and Camargo Jr, C. A. (2017), "Contextual factors associated with hospitals’ decision to operate freestanding emergency departments", *Health Care Management Review,* Vol. 42 No. 3, pp. 269-279.

Patrick, J. and Puterman, M. L. (2008), "Reducing wait times through operations research: optimizing the use of surge capacity", *Healthcare Policy,* Vol. 3 No. 3, p. 75.

Peters, D. H., Adam, T., Alonge, O., Agyepong, I. A. and Tran, N. (2013), "Implementation research: what it is and how to do it", *BMJ,* Vol. 347, p. f6753.

Plantier, M., Havet, N., Durand, T., Caquot, N., Amaz, C., Biron, P., Philip, I. and Perrier, L. (2017a), "Does adoption of electronic health records improve the quality of care management in France? Results from the French e-SI (PREPS-SIPS) study", *International Journal of Medical Informatics,* Vol. 102, pp. 156-165.

Plantier, M., Havet, N., Durand, T., Caquot, N., Amaz, C., Philip, I., Biron, P. and Perrier, L. (2017b), "Does adoption of electronic health records improve organizational performances of hospital surgical units? Results from the French e-SI (PREPS-SIPS) study", *International Journal of Medical Informatics,* Vol. 98, pp. 47-55.

Player, S. (1998), "Activity-based analyses lead to better decision making", *Healthcare Financial Management,* Vol. 52 No. 8, pp. 66-71.

Pomey, M.-P., Forest, P.-G., Sanmartin, C., DeCoster, C., Clavel, N., Warren, E., Drew, M. and Noseworthy, T. (2013), "Toward systematic reviews to understand the determinants of wait time management success to help decision-makers and managers better manage wait times", *Implementation Science,* Vol. 8 No. 1, p. 61.

Poot, C. C., van der Kleij, R. M., Brakema, E. A., Vermond, D., Williams, S., Cragg, L., van den Broek, J. M. and Chavannes, N. H. (2018), "From research to evidence-informed decision making: a systematic approach", *Journal of Public Health,* Vol. 40 No. suppl_1, pp. i3-i12.

Pope, C., Mays, N. and Popay, J. (2006), "How can we synthesize qualitative and quantitative evidence for healthcare policy-makers and managers?", *Healthcare Management Forum,* Vol. 19 No. 1, pp. 27-31.

Pottenger, B. C., Davis, R. O., Miller, J., Allen, L., Sawyer, M. and Pronovost, P. J. (2016), "Comprehensive unit-based safety program (CUSP) to improve patient experience: How a hospital enhanced care transitions and discharge processes", *Quality Management in Healthcare,* Vol. 25 No. 4, pp. 197-202.

Qin, Y., Zhou, R., Wu, Q., Huang, X., Chen, X., Wang, W., Wang, X., Xu, H., Zheng, J. and Qian, S. (2017), "The effect of nursing participation in the design of a critical care information system: a case study in a Chinese hospital", *BMC Medical Informatics and Decision Making,* Vol. 17 No. 1, p. 165.

Råholm, M.-B. (2009), "Evidence and leadership", *Nursing Administration Quarterly,* Vol. 33 No. 2, pp. 168-173.

Ranasinghe, K. I., Chan, T. and Yaralagadda, P. (2012), "Information support for health management in regional Sri Lanka: Health managers' perspectives", *Health Information Management Journal,* Vol. 41 No. 3, pp. 20-26.

Repplinger, M. D., Ravi, S., Lee, A. W., Svenson, J. E., Sharp, B., Bauer, M. and Hamedani, A. G. (2017), "The impact of an emergency department front-end redesign on patient-reported satisfaction survey results", *Western Journal of Emergency Medicine,* Vol. 18 No. 6, p. 1068.

Respicio, A., Moz, M., Pato, M. V., Somensi, R. and Flores, C. D. (2018), "A computational application for multi-skill nurse staffing in hospital units", *BMC Medical Informatics and Decision Making,* Vol. 18 No. 1, p. 53.

Richardson, D. B., Brockman, K., Abigail, A. and Hollis, G. J. (2017), "Effects of a hospital‐wide intervention on emergency department crowding and quality: A prospective study", *Emergency Medicine Australasia,* Vol. 29 No. 4, pp. 415-420.

Richer, M.-C., Dawes, M. and Marchionni, C. (2013), "Bringing knowledge to action in the context of a major organizational transition", *The Health Care Manager,* Vol. 32 No. 1, pp. 4-12.

Robbins, J. and McAlearney, A. S. (2016), "Encouraging employees to speak up to prevent infections: opportunities to leverage quality improvement and care management processes", *American Journal of Infection Control,* Vol. 44 No. 11, pp. 1224-1230.

Ruland, C. (2001), "Developing a decision support system to meet nurse managers' information needs for effective resource management", *Computers in Nursing,* Vol. 19 No. 5, pp. 187-193.

Rundall, T. G., Martelli, P. F., Arroyo, L. and McCurdy, R. (2007), "The informed decisions toolbox: tools for knowledge transfer and performance improvement/practitioner application", *Journal of Healthcare Management,* Vol. 52 No. 5, pp. 325-342.

Sadler, B. L., DuBose, J. and Zimring, C. (2008), "The business case for building better hospitals through evidence-based design", *HERD: Health Environments Research & Design Journal,* Vol. 1 No. 3, pp. 22-39.

Sarkies, M. N., Bowles, K.-A., Skinner, E. H., Haas, R., Lane, H. and Haines, T. P. (2017), "The effectiveness of research implementation strategies for promoting evidence-informed policy and management decisions in healthcare: a systematic review", *Implementation Science,* Vol. 12 No. 1, p. 132.

Schachner, B., González, Z., Cano, R., Luna, D. and Benítez, S. (2017), "Looking for the best WOW: Understanding the nurses’ needs", *MEDINFO 2017: Precision Healthcare through Informatics*, pp. 212-215.

Schaeffer, C., Booton, L., Halleck, J., Studeny, J. and Coustasse, A. (2017), "Big data management in US hospitals: benefits and barriers", *The Health Care Manager,* Vol. 36 No. 1, pp. 87-95.

Seifan, A. and Shemer, J. (2005), "Economic evaluation of medical technologies", *IMAJ,* Vol. 7 No. 2, pp. 67-70.

Sheng, M. L., Chang, S.-Y., Teo, T. and Lin, Y.-F. (2013), "Knowledge barriers, knowledge transfer, and innovation competitive advantage in healthcare settings", *Management Decision,* Vol. 51 No. 3, pp. 461-478.

Shingler-Nace, A. and Gonzalez, J. Z. (2017), "EBM: A pathway to evidence-based nursing management", *Nursing2019,* Vol. 47 No. 2, pp. 43-46.

Shoemaker, L. K., Kazley, A. S. and White, A. (2010), "Making the case for evidence-based design in healthcare: A descriptive case study of organizational decision making", *HERD: Health Environments Research & Design Journal,* Vol. 4 No. 1, pp. 56-88.

Siddharthan, K., Jones, W. J. and Johnson, J. A. (1996), "A priority queuing model to reduce waiting times in emergency care", *International Journal of Health Care Quality Assurance,* Vol. 9 No. 5, pp. 10-16.

Simonen, O., Viitanen, E. and Blom, M. (2012), "Factors relating to effectiveness data use in healthcare management", *International Journal of Productivity and Performance Management,* Vol. 61 No. 7, pp. 752-764.

Soomro, Z. A., Ahmed, J., Muhammad, R., Hayes, D. and Shah, M. H. (2018), "Critical success factors in implementing an e-rostering system in a healthcare organisation", *Health Services Management Research,* Vol. 31 No. 3, pp. 130-137.

Spiers, J. A., Lo, E., Hofmeyer, A. and Cummings, G. G. (2016), "Nurse leaders’ perceptions of influence of organizational restructuring on evidence-informed decision-making", *Nursing Leadership,* Vol. 29 No. 2, pp. 64-81.

Steege, L. M. and Dykstra, J. G. (2016), "A macroergonomic perspective on fatigue and coping in the hospital nurse work system", *Applied Ergonomics,* Vol. 54, pp. 19-26.

Steege, L. M., Pinekenstein, B. J., Arsenault Knudsen, É. and Rainbow, J. G. (2017), "Exploring nurse leader fatigue: a mixed methods study", *Journal of nursing management,* Vol. 25 No. 4, pp. 276-286.

Stelson, P., Hille, J., Eseonu, C. and Doolen, T. (2017), "What drives continuous improvement project success in healthcare?", *International Journal of Health Care Quality Assurance,* Vol. 30 No. 1, pp. 43-57.

Steuten, L. and Buxton, M. (2010), "Economic evaluation of healthcare safety: which attributes of safety do healthcare professionals consider most important in resource allocation decisions?", *Qual Saf Health Care,* Vol. 19 No. 5, pp. e6-e6.

Testik, Ö. M., Shaygan, A., Dasdemir, E. and Soydan, G. (2017), "Selecting health care improvement projects: A methodology integrating cause-and-effect diagram and analytical hierarchy process", *Quality Management in Healthcare,* Vol. 26 No. 1, pp. 40-48.

Thornhill, J., Judd, M. and Clements, D. (2009), "CHSRF knowledge transfer:(re) introducing the self-assessment tool that is helping decision-makers assess their organization's capacity to use research", *Healthcare Quarterly,* Vol. 12 No. 1, p. 22.

Tibor, L. C., Schultz, S. R., Cravath, J. L., Rein, R. R., Krecke, K. N., Baron, M., Joslin, S., Kim, J. S., Shet, N. S. and Pocta, B. (2016), "Improving patient flow utilizing a collaborative learning model", *Radiology management,* Vol. 38 No. 3, pp. 19-28.

Treweek, S., Oxman, A. D., Alderson, P., Bossuyt, P. M., Brandt, L., Brożek, J., Davoli, M., Flottorp, S., Harbour, R. and Hill, S. (2013), "Developing and evaluating communication strategies to support informed decisions and practice based on evidence (DECIDE): protocol and preliminary results", *Implementation Science,* Vol. 8 No. 1, p. 6.

Tricco, A. C., Zarin, W., Rios, P., Straus, S. E. and Langlois, E. V. (2016), "Barriers, facilitators, strategies and outcomes to engaging policymakers, healthcare managers and policy analysts in knowledge synthesis: a scoping review protocol", *BMJ open,* Vol. 6 No. 12, p. e013929.

Ulrich, R. S., Berry, L. L., Quan, X. and Parish, J. T. (2010), "A conceptual framework for the domain of evidence-based design", *HERD: Health Environments Research & Design Journal,* Vol. 4 No. 1, pp. 95-114.

Venugopal, D., Rafi, A. M., Innah, S. J. and Puthayath, B. T. (2017), "Evaluation of process excellence tools in improving donor flow management in a tertiary care hospital in South India", *Asian Journal of Transfusion Science,* Vol. 11 No. 2, p. 135.

Vissers, J. M. (1995), "Patient flow based allocation of hospital resources", *Mathematical Medicine and Biology: A Journal of the IMA,* Vol. 12 No. 3-4, pp. 259-274.

Wallingford Jr, G., Joshi, N., Callagy, P., Stone, J., Brown, I. and Shen, S. (2018), "Introduction of a horizontal and vertical split flow model of emergency department patients as a response to overcrowding", *Journal of Emergency Nursing,* Vol. 44 No. 4, pp. 345-352.

Walshe, K. and Rundall, T. G. (2001), "Evidence‐based management: from theory to practice in health care.", *Milbank Quarterly,* Vol. 79 No. 3, pp. 429-457.

Walston, S. L., Kimberly, J. R. and Burns, L. R. (2001), "Institutional and economic influences on the adoption and extensiveness of managerial innovation in hospitals: The case of reengineering", *Medical Care Research and Review,* Vol. 58 No. 2, pp. 194-228.

Ward Casscells, S., Granger, E., Williams, T. V., Kurmel, T., May, L., Babeau, L., Boyd, D., Davis, D., Ayine, S. and Thomas, N. (2009), "TRICARE management activity healthcare facility evidence-based design survey", *Military medicine,* Vol. 174 No. 3, pp. 236-240.

White, B. A., Yun, B. J., Lev, M. H. and Raja, A. S. (2017), "Applying systems engineering reduces radiology transport cycle times in the emergency department", *Western Journal of Emergency Medicine,* Vol. 18 No. 3, p. 410.

Wiler, J. L., Ozkaynak, M., Bookman, K., Koehler, A., Leeret, R., Chua-Tuan, J., Ginde, A. A. and Zane, R. (2016), "Implementation of a front-end split-flow model to promote performance in an urban academic emergency department", *The Joint Commission Journal on Quality and Patient Safety,* Vol. 42 No. 6, p. 271.

Willems, J. and Ingerfurth, S. (2018), "The quality perception gap between employees and patients in hospitals", *Health Care Management Review,* Vol. 43 No. 2, pp. 157-167.

Williams, L. L. (2006), "What goes around comes around: evidence‐based management", *Nursing Administration Quarterly,* Vol. 30 No. 3, pp. 243-251.

Wills, M. J. (2014), "Decisions through data: Analytics in healthcare", *Journal of Healthcare Management,* Vol. 59 No. 4, pp. 254-262.

Wilson, M., Lavis, J. and Grimshaw, J. (2012), "Supporting the use of research evidence in the Canadian health sector", *Healthcare Q,* Vol. 15, pp. 58-62.

Xie, Z. and Or, C. (2017), "Associations between waiting times, service times, and patient satisfaction in an endocrinology outpatient department: A time study and questionnaire survey", *INQUIRY: The Journal of Health Care Organization, Provision, and Financing,* Vol. 54, p. 0046958017739527.

Yoder, L. (2008), "Evidence-based design", *Nursing Management,* Vol. 39 No. 12, pp. 26-29.

Young, S. K. (2002), "Evidence‐based management: a literature review", *Journal of Nursing Management,* Vol. 10 No. 3, pp. 145-151.

Yu-N, C. and Abidi, S. S. R. (1999), "Healthcare knowledge management through building and operationalising healthcare enterprise memory", *Studies in health technology and informatics,* Vol. 68, pp. 726-730.

Yurumezoglu, H. A. and Kocaman, G. (2012), "Pilot study for evidence‐based nursing management: Improving the levels of job satisfaction, organizational commitment, and intent to leave among nurses in Turkey", *Nursing & Health Sciences,* Vol. 14 No. 2, pp. 221-228.

Zafar, A. M., Suri, R., Nguyen, T. K., Petrash, C. C. and Fazal, Z. (2016), "Understanding preprocedure patient flow in IR", *Journal of Vascular and Interventional Radiology,* Vol. 27 No. 8, pp. 1189-1194.

Zborowsky, T. and Bunker-Hellmich, L. (2010), "Integrating healthcare design research into practice: Setting a new standard of practice", *HERD: Health Environments Research & Design Journal,* Vol. 4 No. 1, pp. 115-130.

Zwijnenberg, N. C., Hendriks, M., Delnoij, D. M., de Veer, A. J., Spreeuwenberg, P. and Wagner, C. (2016), "Understanding and using quality information for quality improvement: the effect of information presentation", *International Journal for Quality in Health Care,* Vol. 28 No. 6, pp. 689-697.
