# Supplementary material for: The neglected contexts and outcomes of evidence-based management: a systematic scoping review in hospital settings
Source: J Health Organ Manag. 2021 Dec 28;36(9):48–65. doi: 10.1108/JHOM-03-2021-0101 (PMC9627724; doi:10.1108/JHOM-03-2021-0101)
Supplement: Supplementary file 3 [file JHOM-03-2021-0101_suppl3.docx]

**Supplementary File 3. Categorization of Articles under the Process of Evidence-based Decision-Making Dimension**

| **Process of Evidence-based Decision-Making Dimension** | | | | |
| --- | --- | --- | --- | --- |
| **Theme** | **Sub-Theme** | | **Articles** | **Example Research** |
| **Acquiring Evidence** | ***Tools for Acquisition*** | | Abidi (1999) | - Abidi (1999) proposed a software that uses data mining as a tool to derive knowledge for from healthcare databases to use in decision-making. - Chan *et al.* (2004) proposed strategies to help managers’ retrieve systematic reviews. - Kibbe *et al.* (1997) proposed a multi-step process and search strategy to guide management researchers, students, and practitioners searching for best practice information on the internet |
|  |  |  | Atack *et al.* (2010) |  |
|  |  |  | Chan *et al.* (2004) |  |
|  |  |  | Davidson (2017) |  |
|  |  |  | Devine *et al.* (2008) |  |
|  |  |  | Doods (2005) |  |
|  |  |  | Kibbe *et al.* (1997) |  |
|  |  |  | Marshall (2013) |  |
|  |  |  | Mathew (2011) |  |
|  |  |  | Ozyapici and Tanis (2016) |  |
|  |  |  | Player (1998) |  |
|  |  |  | Testik *et al.* (2017) |  |
|  | ***Applied Cases***  *Patient flow* | | Aguado-Correa *et al.* (2016) | - Brady *et al.* (2017) described acquiring evidence about the nature of out-of-hours communication between nurses and doctors in a hospital in Ireland. - Elamir (2018) described acquiring organizational evidence about the cause of overcrowding and increased patient length of stay in an Emergency Department in a hospital in Kuwait. |
|  |  |  | Elamir (2018) |  |
|  |  |  | Zafar *et al.* (2016) |  |
|  | *Facility design* | | Mazur *et al.* (2017) |  |
|  | *Medication management* | | Debono *et al.* (2017) |  |
|  | *Patient experience* | | Xie and Or (2017) |  |
|  | *Policy* | | Mahmoudian-Dehkordi and Sadat (2017) |  |
|  | *Team communication* | | Brady *et al.* (2017) |  |
|  | ***Acquiring Evidence about a Problem***  *Nursing burnout & fatigue* | | Furnham *et al.* (2003) | - Attree (2001) conducted a qualitative study to acquire evidence about how nurses, doctors, managers, patient, and relatives describe quality of care and what criteria they use to evaluate it. - Bucci *et al.* (2016) conducted a systematic review to acquired evidence about how Lean Management methods and tools can be applied to address the problem of overcrowding in Emergency Departments. - Jiang and Verderber (2017) conducted a systematic review to acquired evidence about the relationship between the design of hospital circulation zone (i.e. corridors, lobbies, elevators) and health-related outcomes (i.e. stress, satisfaction, patient falls). - Myers *et al.* (2016) conducted a qualitative study to acquire evidence about nurses’ experiences of horizontal violence (i.e. hostile behavior by nurses towards nurses) in different types of hospitals. |
|  |  |  | Myers *et al.* (2016) |  |
|  |  |  | Nantsupawat *et al.* (2017) |  |
|  |  |  | Steege and Dykstra (2016) |  |
|  |  |  | Steege *et al.* (2017) |  |
|  | *Quality & safety* | | Attree (2001) |  |
|  |  |  | DeWulf *et al.* (2017) |  |
|  |  |  | Finkelstein *et al.* (1997) |  |
|  |  |  | Steuten and Buxton (2010) |  |
|  |  |  | Willems and Ingerfurth (2018) |  |
|  | *Wait Time and Crowding* | | Bucci *et al.* (2016) |  |
|  |  |  | Khalifa and Zabani (2016a) |  |
|  |  |  | Kreindler (2008) |  |
|  |  |  | Pomey *et al.* (2013) |  |
|  | *Facility design* | | Ibrahim *et al.* (2017) |  |
|  |  |  | Jiang and Verderber (2017) |  |
|  |  |  | Ward Casscells *et al.* (2009) |  |
|  | *EBMgt* | | Guo *et al.* (2017) |  |
|  | *Freestanding emergency department* | | Patidar *et al.* (2017). |  |
|  | *Knowledge translation* | | Gagliardi and Dobrow (2016) |  |
|  | *Lean implementation* | | Al-Hyari *et al.* (2016) |  |
|  | *Medication management* | | Härkänen *et al.* (2017) |  |
|  | *Patient experience* | | Bendesky *et al.* (2016) |  |
|  | *Performance assessment* | | Fanelli and Zangrandi (2017) |  |
|  | *Resource deployment* | | Chow *et al.* (1999) |  |
|  | *Value-based healthcare* | | Nilsson *et al.* (2017) |  |
| **Appraising the Quality of the Evidence** | ***Analyzing Data*** | |  | Delias *et al.* (2015) introduced a method to analyze emergency department process data to support decision making. |
|  | ***Strategies & Tools for Appraisal*** | | Davidson (2017) | Lohr (2004) classified and evaluated existing systems for grading the quality of research articles and bodies of evidence. |
|  |  |  | Kibbe *et al.* (1997) |  |
|  |  |  | Lohr (2004) |  |
| **Generating**  **Alternatives** | | | Elamir (2018) | Elamir (2018) described how several evidence-based alternates were generated using organizational data and scientific literature to solve overcrowding in a hospital Emergency Department in Kuwait. |
| **Making a Decision** | |  | Testik *et al.* (2017) | Testik *et al.* (2017) developed and tested a tool for objectively choosing between different alternatives. |
| **Preparing for Implementation** | | ***Factors Impacting Implementation*** | Gallego *et al.* (2008) | - Pomey *et al.* (2013) conducted a systematic review on frameworks that can be used to analyze factors that influence the success of waiting time management strategies. - Soomro *et al.* (2018) examined the factors that contribute to successful implementation of an e-roistering system in a hospital. |
|  |  |  | Korlén *et al.* (2017) |  |
|  |  |  | Pomey *et al.* (2013) |  |
|  |  |  | Robbins and McAlearney (2016) |  |
|  |  |  | Soomro *et al.* (2018) |  |
|  |  |  | Stelson *et al.* (2017) |  |
|  |  |  | Walston *et al.* (2001) |  |
|  |  | ***Strategies & Tools for Implementation*** | Gignon *et al.* (2017) | Gignon *et al.* (2017) discuss how simulation can be used to design, plan, and assess a new hospital building before opening it for patient care. |
|  |  |  | Guzman *et al.* (2015) |  |
|  |  |  | Newhouse and White (2011) |  |
|  |  | ***Applied Cases***  *Facility Design* | Chiarini and Baccarani (2016) | Mazur *et al.* (2017) described how they applied lean management principles and strategies during the design phase of a new surgery building. |
|  |  |  | Johnson *et al.* (2017) |  |
|  |  |  | Mazur *et al.* (2017) |  |
| **Assessing & Adjusting** | | ***Applied Cases***  *Admission* | Jessup *et al.* (2016) | - Büchner *et al.* (2016) assessed the impact of entering a health system on hospital efficiency and profitability in hospitals in Germany. - de-Carvalho *et al.* (2017) assessed the implementation of an automated drug-dispensing system on errors in drug administration in a hospital in Brazil. - Karliner *et al.* (2017) assessed the impact of having easy access to professional interpreters at each hospital bedside on readmission rates, length of stay, and hospital expenditures in USA. - Plantier and colleagues (2017b, 2017a) assessed the implementation of Electronic Health Record on the performance of surgical units and overall quality of care in hospitals throughout France. - Repplinger *et al.* (2017) assessed the impact of redesigning the emergency department front-end on patient satisfaction scores in a hospital in USA. |
|  |  |  | Karliner *et al.* (2017) |  |
|  |  | *Bed Allocation* | Doorduijn *et al.* (2016) |  |
|  |  |  | Novati *et al.* (2017) |  |
|  |  | *Discharge* | Holland *et al.* (2017) |  |
|  |  |  | Luo *et al.* (2017) |  |
|  |  | *Electronic health records* | Plantier *et al.* (2017a) |  |
|  |  |  | Plantier *et al.* (2017b) |  |
|  |  | *Facility Design* | Donetto *et al.* (2017) |  |
|  |  |  | Krugman *et al.* (2015) |  |
|  |  | *Patient experience* | Kowalski *et al.* (2017) |  |
|  |  |  | Repplinger *et al.* (2017) |  |
|  |  | *Patient flow* | Naidoo and Mahomed (2016) |  |
|  |  |  | Richardson *et al.* (2017) |  |
|  |  | *Staffing* | Claret *et al.* (2016) |  |
|  |  |  | Maass *et al.* (2017) |  |
|  |  | *Accreditation* | Chen *et al.* (2016) |  |
|  |  | *Foodservices* | Büchner *et al.* (2016) |  |
|  |  | *Forecasting* | Schachner *et al.* (2017) |  |
|  |  | *Health systems* | de-Carvalho *et al.* (2017) |  |
|  |  | *Medical Equipment* | Chiarini and Baccarani (2016) |  |
|  |  | *Medication Management* | Buttigieg *et al.* (2016) |  |
|  |  | *Performance Improvement* | Foglia *et al.* (2017) |  |
|  |  | *Quality Improvement* | Chen *et al.* (2016) |  |
|  |  | *Technology Assessment* | Doorduijn *et al.* (2016). |  |
| **Overall Process** | | ***Mapping the Process*** | Baghbanian *et al.* (2012) | Brown and Ecoff (2011), through a conceptual study, proposed an eight-step approach to evidence-based decision making in the context of healthcare facility design. |
|  |  |  | Brown and Ecoff (2011) |  |
|  |  |  | Gallego *et al.* (2008) |  |
|  |  |  | Janati *et al.* (2018) |  |
|  |  |  | Oetjen *et al.* (2008) |  |
|  |  | ***Support Tools*** | Fernandez *et al.* (1997) | Gartnera and Padmanb (2017) developed a digital workbench for hospital resource planning decisions. |
|  |  |  | Gartnera and Padmanb (2017) |  |
|  |  | ***Applied Cases***  *Forecasting* | Afilal *et al.* (2016) | - Fulbrook *et al.* (2017) proposed and assessed having nurse navigators to facilitate patient movement through the Emergency Department on throughput in a hospital in Australia. - Hicks *et al.* (2017) designed and tested a quality improvement initiative to reduce unnecessary blood transfusions in the Department of Surgery of a hospital in USA. - Khalifa (2017) conducted a root cause analysis to identify reasons for delays in discharging inpatients. They then designed and launched a performance improvement project, which involved collecting data, applying several interventions, and assessing their impact on discharge and average length of stay in a hospital in the Kingdom of Saudi Arabia. - Lucini *et al.* (2017) proposed and tested a Text Mining approach to analyze free-text medical records from Emergency Department patients soon after they make first contact with Emergency Department physicians to better predict admission in a hospital in Brazil. - Qin *et al.* (2017) actively involved nurses in the design, development, and implementation of an intensive care information system and examined the impact on nursing care processes and nurse satisfaction in a hospital in China. - White *et al.* (2017) applied and tested a series of process improvement interventions based on lean methodologies to address delays in radiology test turnaround time in an Emergency department of a hospital in USA. |
|  |  |  | Barak-Corren *et al.* (2017) |  |
|  |  |  | Calegari *et al.* (2016) |  |
|  |  |  | Lucini *et al.* (2017) |  |
|  |  |  | Parente *et al.* (2018) |  |
|  |  | *Patient flow* | Fulbrook *et al.* (2017) |  |
|  |  |  | Lovett *et al.* (2016) |  |
|  |  |  | Tibor *et al.* (2016) |  |
|  |  |  | Venugopal *et al.* (2017) |  |
|  |  |  | Wiler *et al.* (2016) |  |
|  |  | *Information system* | Krugman and Sanders (2016) |  |
|  |  |  | Nippak *et al.* (2016) |  |
|  |  |  | Qin *et al.* (2017) |  |
|  |  |  | Ruland (2001) |  |
|  |  | *Patient experience* | Bellamkonda *et al.* (2016) |  |
|  |  |  | Gillespie and Reader (2016) |  |
|  |  |  | Nelson and Staffileno (2017) |  |
|  |  |  | Pottenger *et al.* (2016) |  |
|  |  | *Process improvement* | Bell *et al.* (2016) |  |
|  |  |  | Bowen *et al.* (2016) |  |
|  |  |  | Hicks *et al.* (2017) |  |
|  |  |  | Khalifa and Zabani (2016b) |  |
|  |  | *Staffing* | Butler *et al.* (2012) |  |
|  |  |  | DeRienzo *et al.* (2017) |  |
|  |  |  | Kullberg *et al.* (2016) |  |
|  |  |  | Respicio *et al.* (2018) |  |
|  |  | *Emergency department crowding* | Eiset *et al.* (2016) |  |
|  |  |  | Siddharthan *et al.* (1996) |  |
|  |  |  | Wallingford Jr *et al.* (2018) |  |
|  |  | *Bed allocation* | da Silveira Grübler *et al.* (2018) |  |
|  |  |  | Vissers (1995) |  |
|  |  | *Quality improvement* | Gold *et al.* (2016) |  |
|  |  |  | Nates *et al.* (2017) |  |
|  |  | *Test turnaround time* | Inal *et al.* (2018) |  |
|  |  |  | White *et al.* (2017) |  |
|  |  | *Employee Satisfaction* | Yurumezoglu and Kocaman (2012) |  |
|  |  | *Hospital design* | Yoder (2008) |  |
|  |  | *Patient discharge* | Khalifa (2017) |  |
|  |  | *Policy selection* | Carnero and Gómez (2016) |  |
|  |  | *Priority setting* | Astley and Wake-Dyster (2001) |  |
