# Supplementary material for: The neglected contexts and outcomes of evidence-based management: a systematic scoping review in hospital settings
Source: J Health Organ Manag. 2021 Dec 28;36(9):48–65. doi: 10.1108/JHOM-03-2021-0101 (PMC9627724; doi:10.1108/JHOM-03-2021-0101)
Supplement: Supplementary file 4 [file JHOM-03-2021-0101_suppl4.docx]

**Supplementary File 4. Categorization of Articles under the Sources of Evidence Dimension**

| **Sources of Evidence Dimension** | | | |
| --- | --- | --- | --- |
| **Theme** | **Sources Mapped onto Model** | **Articles** | **Example Research** |
| **Concept of evidence** |  | Råholm (2009) | Råholm (2009) argued that the concept of evidence in EBMgt is rigid, portrayed it from a multidimensional perspective, and argued for rethinking it. |
| **Evidence used in Practice** | Experiential, Scientific, Organizational, Stakeholder | Guo *et al.* (2017) | - Guo *et al.* (2017) examined the types of evidence hospital managers across the USA used in their decision-making. - Shoemaker *et al.* (2010) described the different types of evidence used for a facility design decision in a hospital in USA. |
|  |  | Janati *et al.* (2018) |  |
|  |  | Liang *et al.* (2012) |  |
|  |  | Spiers *et al.* (2016) |  |
| ***Applied Cases*** | Experiential, Scientific, Organizational, Stakeholder | Shoemaker *et al.* (2010) |  |
|  | Scientific, Organizational, Stakeholder | Richer *et al.* (2013) |  |
|  | Organizational, Stakeholder | Beglinger (2006) |  |
| **Encouraging the use of a specific type of evidence**          ***Applied Cases*** | Organizational | Ginsburg (2003) | - Capan *et al.* (2017) argued for the use of operations research to inform healthcare delivery decision-making and highlighted potential opportunities for its use. - Hawkins *et al.* (2016) demonstrated their use of Twitter as a supplementary data source to measure patient-perceived quality of care in US hospitals. - Patrick and Puterman (2008) discussed the benefit of using operations research in healthcare management and demonstrated its benefits for optimizing scheduling and reducing reduce wait times. - Schaeffer *et al.* (2017) argued for the use of big data and discussed its potential benefits to hospitals. |
|  |  | Jan (2003) |  |
|  |  | Murphy *et al.* (2013) |  |
|  |  | Mykkänen *et al.* (2016) |  |
|  |  | Schaeffer *et al.* (2017) |  |
|  |  | Simonen *et al.* (2012) |  |
|  | Scientific | Capan *et al.* (2017) |  |
|  |  | Peters *et al.* (2013) |  |
|  | Organizational | Fagerström (2009) |  |
|  |  | Gignon *et al.* (2017) |  |
|  |  | Hawkins *et al.* (2016) |  |
|  |  | Margrif (1991) |  |
|  |  | Seifan and Shemer (2005) |  |
|  |  | Vissers (1995) |  |
|  | Scientific | AbuKhousa *et al.* (2014) |  |
|  |  | Bai *et al.* (2018) |  |
|  |  | Patrick and Puterman (2008) |  |
