# Supplementary material for: The neglected contexts and outcomes of evidence-based management: a systematic scoping review in hospital settings
Source: J Health Organ Manag. 2021 Dec 28;36(9):48–65. doi: 10.1108/JHOM-03-2021-0101 (PMC9627724; doi:10.1108/JHOM-03-2021-0101)
Supplement: Supplementary file 5 [file JHOM-03-2021-0101_suppl5.docx]

| **Supplementary File 5. Categorization of Articles under the Barriers & Facilitators Dimension** | | | |
| --- | --- | --- | --- |
|  | | | |
| **Barriers & Facilitators Dimension** | | | |
| **Theme** | **Barriers Mapped onto Model** | **Article** | **Example Research** |
| Examining one Barrier/ Facilitator | Characteristics of the Evidence | Aldrich *et al.* (2006) | - Alexander *et al.* (2007) examined the types of information hospital chief executive officers need to address cost and quality problems and the extent to which health services research is meeting those needs. - Zborowsky and Bunker-Hellmich (2010) discuss evidence-based design decision-making and the challenges and opportunities that exist related to the existing research evidence. - Zwijnenberg *et al.* (2016) examined how information presentation effects the way it is understood and used for quality improvement. |
|  |  | Alexander *et al.* (2007) |  |
|  |  | Atack *et al.* (2010) |  |
|  |  | Bai *et al.* (2018) |  |
|  |  | Barton (1994) |  |
|  |  | Ferlie and Wood (2003) |  |
|  |  | Finkler and Ward (2003) |  |
|  |  | Gautam (2008) |  |
|  |  | Green (2011) |  |
|  |  | Kontio *et al.* (2013) |  |
|  |  | Lomas (2005) |  |
|  |  | Matchar *et al.* (2005) |  |
|  |  | Poot *et al.* (2018) |  |
|  |  | Pope *et al.* (2006) |  |
|  |  | Treweek *et al.* (2013) |  |
|  |  | Tricco *et al.* (2016) |  |
|  |  | Ulrich *et al.* (2010) |  |
|  |  | Zborowsky and Bunker-Hellmich (2010) |  |
|  |  | Zwijnenberg *et al.* (2016) |  |
|  | Characteristics of the Decision Maker | Adams *et al.* (2016) | - Finkler (2002) discuss the necessity of providing research-oriented education as a way to overcome barriers to evidence-based management. - Nicklin and Stipich (2005), described the goals of a program aimed to enhance healthcare executives’ skills in using research for decision making. |
|  |  | Bigelow and Arndt (2003) |  |
|  |  | Browman *et al.* (2003) |  |
|  |  | Burgess and Currie (2013) |  |
|  |  | Finkler (2002) |  |
|  |  | Fischer *et al.* (2016) |  |
|  |  | Fletcher and Thornhill (2009) |  |
|  |  | Jbilou *et al.* (2009) |  |
|  |  | Lavoie‐Tremblay *et al.* (2012a) |  |
|  |  | Nicklin and Stipich (2005) |  |
|  | Organizational Structure & Culture | Allen (1997) | - Ellen *et al.* (2013) examine the different programs, interventions, instruments, and tools that healthcare organizations in Canada have to support evidence- informed decision-making and which are perceived to actually facilitate evidence-based decision-making. - Thornhill *et al.* (2009) discussed a self-assessment tool that can be used by healthcare organizational to examine their capacity to acquire, interpret, and rely on research evidence to make decisions and deliver healthcare services. |
|  |  | Ellen *et al.* (2011) |  |
|  |  | Ellen *et al.* (2013) |  |
|  |  | Finkler (2004) |  |
|  |  | Friedman (1999) |  |
|  |  | Innis and Berta (2016) |  |
|  |  | Jan (2003) |  |
|  |  | Jayakumar *et al.* (2016) |  |
|  |  | Kovner *et al.* (2001) |  |
|  |  | Lavoie‐Tremblay *et al.* (2012b) |  |
|  |  | Murphy *et al.* (2013) |  |
|  |  | Ouimet *et al.* (2014) |  |
|  |  | Rundall *et al.* (2007) |  |
|  |  | Sarkies *et al.* (2017) |  |
|  |  | Thornhill *et al.* (2009) |  |
|  |  | Wills (2014) |  |
|  |  | Yu-N and Abidi (1999) |  |
|  | National Structure & Culture | Mykkänen *et al.* (2016) | Wilson *et al.* (2012) documented five Canadian contributions to facilitate the acquisition and use of research evidence. |
|  |  | Ranasinghe *et al.* (2012) |  |
|  |  | Wilson *et al.* (2012) |  |
| Examining overall Barriers & Facilitators | - Characteristics of the Evidence - Characteristics of the Decision Maker | Axelsson (1998) | - Ellen *et al.* (2014) identified the barriers and facilitators to implementing programs, interventions, instruments, and tools to support evidence- informed decision-making in Canadian healthcare organizations. - Golenko *et al.* (2012) assessed the barriers and facitlaitros to research capacity building from the perspective of senior healthcare managers in allied health. - Guo *et al.* (2017) examined the influence of managers’ demographic characteristics and attitudes, and the size of the healthcare organization on healthcare managers’ use of evidence-based management. - Karamitri *et al.* (2017) systematically reviewed the literature on knowledge management in healthcare settings and identified the barriers to implementation of knowledge management. - Kovner and Rundall (2006) suggest practical strategies that healthcare organizations in USA can adopt to strengthen the implementation of evidence-based management. - Liang *et al.* (2012)  conducted a systematic review on evidence- informed decision-making, including the barriers to its practice among health service managers. - Niedzwiedzka (2003) examined the individual and environmental factors that influence healthcare managers’ information use in hospitals in Poland. |
|  |  | Gallego *et al.* (2008) |  |
|  |  | Kovner *et al.* (2000) |  |
|  |  | Sheng *et al.* (2013) |  |
|  | - Characteristics of the Evidence - Organizational Structure & Culture | Gagliardi and Dobrow (2016) |  |
|  |  | Ginsburg (2003) |  |
|  |  | Golenko *et al.* (2012) |  |
|  |  | Jih *et al.* (2006) |  |
|  |  | Marshall (2013) |  |
|  | - Characteristics of the Evidence - National Structure & Culture | Clancy and Cronin (2005) |  |
|  |  | Leatherman and Sutherland (2007) |  |
|  | - Characteristics of the Evidence - Characteristics of the Decision Maker - Organizational Structure & Culture | Ellen *et al.* (2014) |  |
|  |  | Jbilou *et al.* (2007) |  |
|  |  | Karamitri *et al.* (2017) |  |
|  |  | Langaneer and Worthington (2010) |  |
|  |  | Niedzwiedzka (2003) |  |
|  |  | Simonen *et al.* (2012) |  |
|  | - Characteristics of the Evidence - Characteristics of the Decision Maker - National Structure & Culture | Liang *et al.* (2012) |  |
|  | - Characteristics of the Decision Maker - Organizational Structure & Culture | Guo *et al.* (2017) |  |
|  |  | Champagne *et al.* (2014) |  |
|  |  | Janati *et al.* (2018) |  |
|  |  | Shoemaker *et al.* (2010) |  |
|  |  | Spiers *et al.* (2016) |  |
|  |  | Williams (2006) |  |
|  | - Organizational Structure & Culture - National Structure & Culture | Canaway *et al.* (2017) |  |
|  | - Characteristics of the Evidence - Characteristics of the Decision Maker - Organizational Structure & Culture - National Structure & Culture | Kovner and Rundall (2006) |  |
|  |  | Walshe and Rundall (2001) |  |
