# Supplementary material for: The neglected contexts and outcomes of evidence-based management: a systematic scoping review in hospital settings
Source: J Health Organ Manag. 2021 Dec 28;36(9):48–65. doi: 10.1108/JHOM-03-2021-0101 (PMC9627724; doi:10.1108/JHOM-03-2021-0101)
Supplement: Supplementary file 6 [file JHOM-03-2021-0101_suppl6.docx]

**Supplementary File 6. Categorization of Articles under the Decision Criteria Dimension**

| **Decision Criteria Dimension** | | | |
| --- | --- | --- | --- |
| **Theme** | **Decision Criteria Mapped onto Model** | **Articles** | **Example Research** |
| **Identifying Criteria** | Organizational | Friedman (1999) | Spiers *et al.* (2016) examined nurse leaders’ evidence-based decision-making process in a context of continuous restructuring, and found that political and fiscal criteria inherent in system restructuring took precedent over patient needs. |
|  | Organizational, External | Spiers *et al.* (2016) |  |
|  | Organizational, Stakeholder | Shoemaker *et al.* (2010) |  |
|  | Organizational, Stakeholder, External | Gallego *et al.* (2008) |  |
|  | Ethico-Legal | Janati *et al.* (2018) |  |
| **Pinpointing Criteria in the Process** | - | Oetjen *et al.* (2008) | Oetjen *et al.* (2008) offered a conceptually developed evidence-based managerial decision-making modal, which included developing and ranking criteria once a problem is identified and then using the criteria to choose between alternatives. |
| **Pinpointing & identifying Criteria in the Process** | Organizational, Ethico-Legal, Technical | Baghbanian *et al.* (2012) | Baghbanian *et al.* (2012) empirically developed a decision-making model for resource allocation, which depicted different decision criteria that are used to evaluate possible positions and create contextual fit. |
|  | Organizational, Stakeholder | Beglinger (2006) |  |
