# Supplementary material for: The neglected contexts and outcomes of evidence-based management: a systematic scoping review in hospital settings
Source: J Health Organ Manag. 2021 Dec 28;36(9):48–65. doi: 10.1108/JHOM-03-2021-0101 (PMC9627724; doi:10.1108/JHOM-03-2021-0101)
Supplement: Supplementary file 7 [file JHOM-03-2021-0101_suppl7.docx]

**Supplementary File 7. Categorization of Articles under the Evidence-based Management Concept**

| **Evidence-based Management Concept** | | | |
| --- | --- | --- | --- |
| **Dimension** | **Theme** |  | **Example Research** |
| **EBMgt Introduction & Promotion** | ***Healthcare Management*** | Axelsson (1998) | - Walshe and Rundall (2001) introduced the core principles of evidence-based management in healthcare, compared it to evidence-based medicine, described its increasing acceptance, and explored the reasons behind it. |
|  |  | Clancy and Cronin (2005) |  |
|  |  | Kovner and Rundall (2006) |  |
|  |  | Kovner *et al.* (2000) |  |
|  |  | Ovretveit (1999) |  |
|  |  | Walshe and Rundall (2001) |  |
|  |  | Young (2002) |  |
| **EBMgt Promotion** | ***Facility Design*** | Berry *et al.* (2004) | - Finkler (2004) argued that healthcare financial management can benefit from the evidence-based management approach and discussed a framework for its application. - Cohen (2011) discussed the financial benefits of using the evidence-based management approach for managing the healthcare workforce. |
|  |  | Sadler *et al.* (2008) |  |
|  |  | Ulrich *et al.* (2010) |  |
|  |  | Zborowsky and Bunker-Hellmich (2010) |  |
|  | ***Financial Management*** | Finkler (2004) |  |
|  |  | Finkler and Ward (2003) |  |
|  | ***Nursing Management*** | Shingler-Nace and Gonzalez (2017) |  |
|  |  | Williams (2006) |  |
|  | ***Health Technology Assessment*** | Juzwishin (2010) |  |
|  | ***Human Resource Management*** | Cohen (2011) |  |
|  | ***Risk Management*** | Card *et al.* (2012) |  |
| **EBMgt Criticism** |  | Hewison (2004) | Hewison (2004) argued that EBMgt is incongruent with current management practice. |
